# Gold Nanoparticles for Photothermal and Photodynamic Therapy

**DOI:** 10.1021/acsomega.4c08797

**Published:** 2024-10-23

**Authors:** Matthew Broadbent, Samantha J. Chadwick, Mathias Brust, Martin Volk

**Affiliations:** Department of Chemistry, University of Liverpool, Crown Street, Liverpool L69 7ZD, U.K.

## Abstract

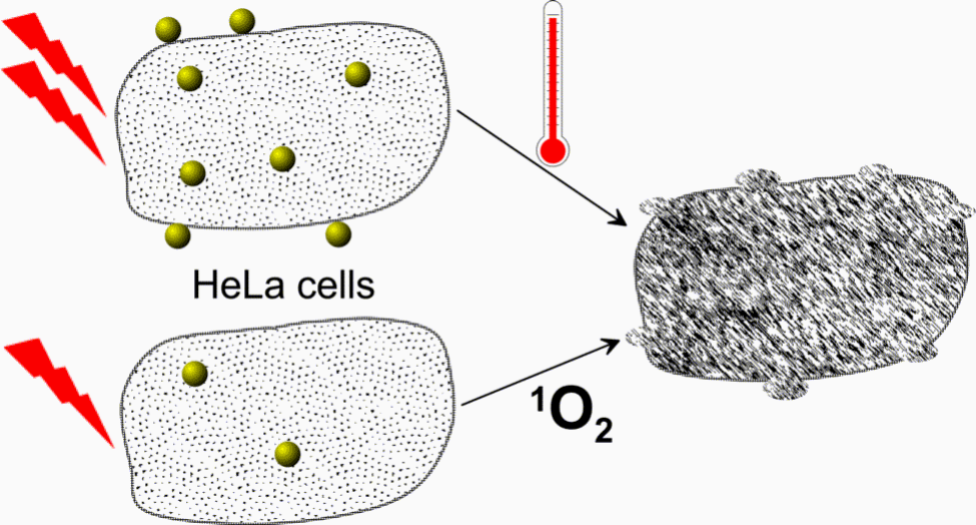

Cancer cells exposed to 13 nm gold nanoparticles and irradiated with cw laser light at 532 nm are shown to undergo cell death via two competing causes. When cells contain relatively high quantities of gold nanoparticles and/or receive a high dose of light, photothermal effects dominate, which are independent of the cellular location of the gold nanoparticles and affect all cells in the irradiated area due to the rapid diffusion of heat. In contrast, at lower doses of nanoparticles and light, the photogeneration of singlet oxygen triggers cell death only in cells that contain a sufficient number of nanoparticles. The parallel occurrence of both effects will need to be considered carefully when designing practical therapy applications. In particular, the photodynamic effect should allow for a cell-type-specific treatment modality that can distinguish between cancer and normal cells using suitable targeting ligands on the nanoparticle surface, providing a highly selective route for cancer therapy.

## Introduction

Nanoparticles show much promise as a key resource in the ever-growing toolkit that clinicians can use to detect or kill cancer cells using minimally invasive approaches. In particular, gold nanoparticles (AuNPs), which may be solid spheres, shells, or have rod- or star-like shapes and usually are biocompatible, have shown promise as photothermal therapy (PTT) agents in work pioneered by El-Sayed and others, which initially focused mostly on in vitro studies,^1−14^ but more recently has also been validated in vivo;^3−9,15^ first human clinical trials have been undertaken with success or are in progress.^3,16,17^ These studies have shown that AuNPs can target the inside of cancerous cells, and then irradiation with light corresponding to the AuNPs’ plasmon band kills the cells via thermal heating. The usefulness of AuNPs for PTT applications is based on their strong absorbance at tunable wavelengths and the subsequent fast conversion of the absorbed light energy into heat.^18^

However, AuNPs have also been shown to generate singlet oxygen upon irradiation, which makes them candidates for their use as photodynamic therapy (PDT) agents.^14,19−25^ PDT utilizes light and a photosensitizer (PS) within tumors to generate reactive oxygen species (ROS), which cause oxidative damage and kill the cell *via* apoptotic, necrotic, or autophagic pathways.^26,27^ The use of AuNPs as PDT-PS would have several clinical advantages over AuNP-mediated PTT: provided that ROS can be photogenerated with sufficient yield, it would require lower concentrations of AuNPs and/or lower light powers, but even more importantly, it could potentially act locally against individual cancerous cells, whereas PTT necessarily acts on larger volumes due to the fast diffusion of heat. AuNPs would present several advantages over organic PSs due to the absence of photobleaching or enzymatic degradation. Moreover, AuNPs can carry multiple functional ligands, which could provide targeting to specific cell types as well as diagnostic properties (fluorescence, surface-enhanced Raman, or even magnetic resonance imaging markers) in addition to their photosensitizing properties.^4−6^

There is considerable confusion in the literature about whether a given treatment modality affects cells via the PTT or PDT mechanism. It is often assumed that PTT constitutes the main mechanism without clear proof, even in situations where the same effect on cells was reported when irradiating them at 4 and 37 °C,^14^ where significant cell death occurred upon heating to 40 °C for less than a minute,^13^ which normally does not lead to cell death (see below), or where a simple estimate shows that all of the incident light would need to be absorbed in a single cell layer and converted to heat to achieve significant heating,^28^ which is not possible given AuNP’s optical cell uptake properties. Similarly, other reports suggest PDT as the relevant mechanism without any attempt at estimating the temperature induced by the irradiation of the AuNPs present or providing some other evidence supporting this assignment and ruling out significant local heating.^19,20^

Here, we show that cells containing AuNPs can be killed via both mechanisms upon irradiation with visible light and that the dominant mechanism can be selected by the use of different AuNP coatings, the number of AuNPs that are internalized within the cells, and the light intensities employed. The outcomes of cell killing by the different mechanisms have significantly different characteristics. These will be important to consider for achieving the optimum treatment in different potential applications of AuNPs in light-induced cancer therapy, and any investigation of such therapy approaches needs to carefully distinguish between PTT and PDT effects.

## Methods

### Nanoparticle Synthesis and Characterization

**Scheme 1:**
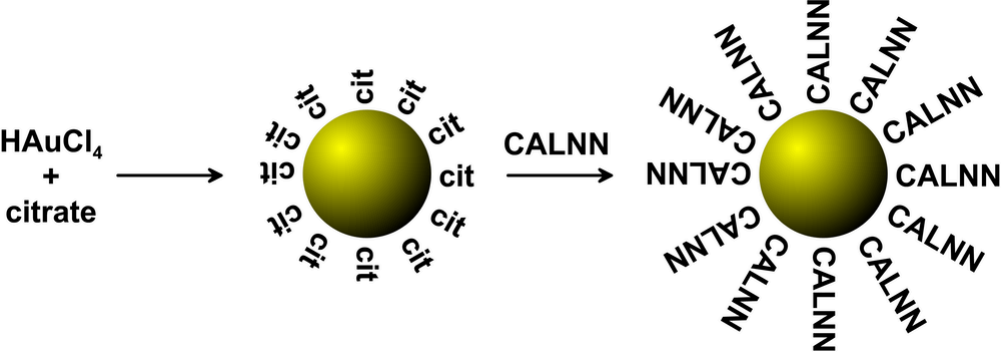
Synthesis and Modification of 13–15 nm AuNPs (Capping Ligand Size Not to Scale)

Monodisperse citrate-stabilized AuNPs with diameters of 13–15 nm were synthesized by standard methods. Covalent capping of AuNPs by a pentapeptide with the sequence CALNN was achieved by overnight incubation. These steps are schematically shown in Scheme 1. All samples were characterized by UV–vis spectroscopy and differential centrifugal sedimentation. More details and typical results are given in the Supporting Information.

### Nanoparticle Uptake by HeLa Cells

HeLa cells were incubated with a 1:1 (v/v) mixture of a cell culture medium (CCM; Dulbecco's modified Eagle’s medium, supplemented with 10% fetal bovine serum) and AuNP solution, at different AuNP concentrations and for different times, under standard growth conditions (37 °C, 5% CO_2_). We did not attempt to increase the concentration of the citrate-stabilized AuNPs after synthesis and hence were limited to an incubation concentration of 2 nM. On the other hand, CALNN-stabilized AuNPs at higher concentrations could easily be prepared at slightly higher concentrations during the cleaning steps following incubation with CALNN, and we observed that a higher incubation concentration was needed to get a comparable uptake of CALNN-stabilized AuNPs into the cell interior, see below, which is why we normally used a concentration of 4 nM. The incubation mixture was prepared 24 h before cell incubation to allow for the reproducible formation of a serum protein corona on the AuNPs, which is required for cell uptake by endocytosis; compare Section S1.5 of the Supporting Information. After repeated washing with phosphate-buffered saline (PBS, pH 7.4), the cells were detached using trypsin, the cells in a small aliquot were counted, and the remaining cells were dissolved in aqua regia. The gold content of the sample was determined using inductively coupled plasma optical emission spectrometry (ICP-OES) and used to calculate the number of AuNPs per cell.

For obtaining (scanning) transmission electron microscopy (TEM/STEM) images of cells after incubation with AuNPs, cells were washed repeatedly with PBS and then embedded in epoxy resin. Ultrathin sections (ca. 70 nm) were cut using a diamond knife, mounted on mesh copper grids, and post-stained with uranyl acetate and lead citrate solutions.

### HeLa Cell Irradiation

HeLa cells were incubated with a 1:1 (v/v) mixture of CCM and AuNP solution following the protocol described above. The cells in the target area were imaged using a microscope and, after repeated washing and immersion in PBS (pH 7.4), irradiated at room temperature with cw laser light at 532 nm. Unless stated otherwise, they were then kept under standard growth conditions in CCM for 24 h, when cell viability was assessed using microscope images after applying trypan blue, which stains cells with a compromised membrane. The number of live cells, identified by the absence of staining or significant morphological changes, was counted in a circular area with a diameter of 0.5 mm around the center of the laser beam, over which the light intensity drops by less than 14%, for the images taken prior to and 24 h after irradiation and used to calculate cell viability by comparing their ratio to the expected growth rate, obtained from a non-irradiated control area on the same dish. A few experiments using the MTT assay as an alternative measure for cell viability are described in Section S8 of the Supporting Information.

More details, including cell culture and irradiation protocols, CCM composition, uptake analysis, TEM sample preparation, and cell viability analysis, are provided in the Supporting Information. Error bars are the standard deviations of repeat experiments.

## Results and Discussion

### Uptake of Gold Nanoparticles

**Figure 1:**
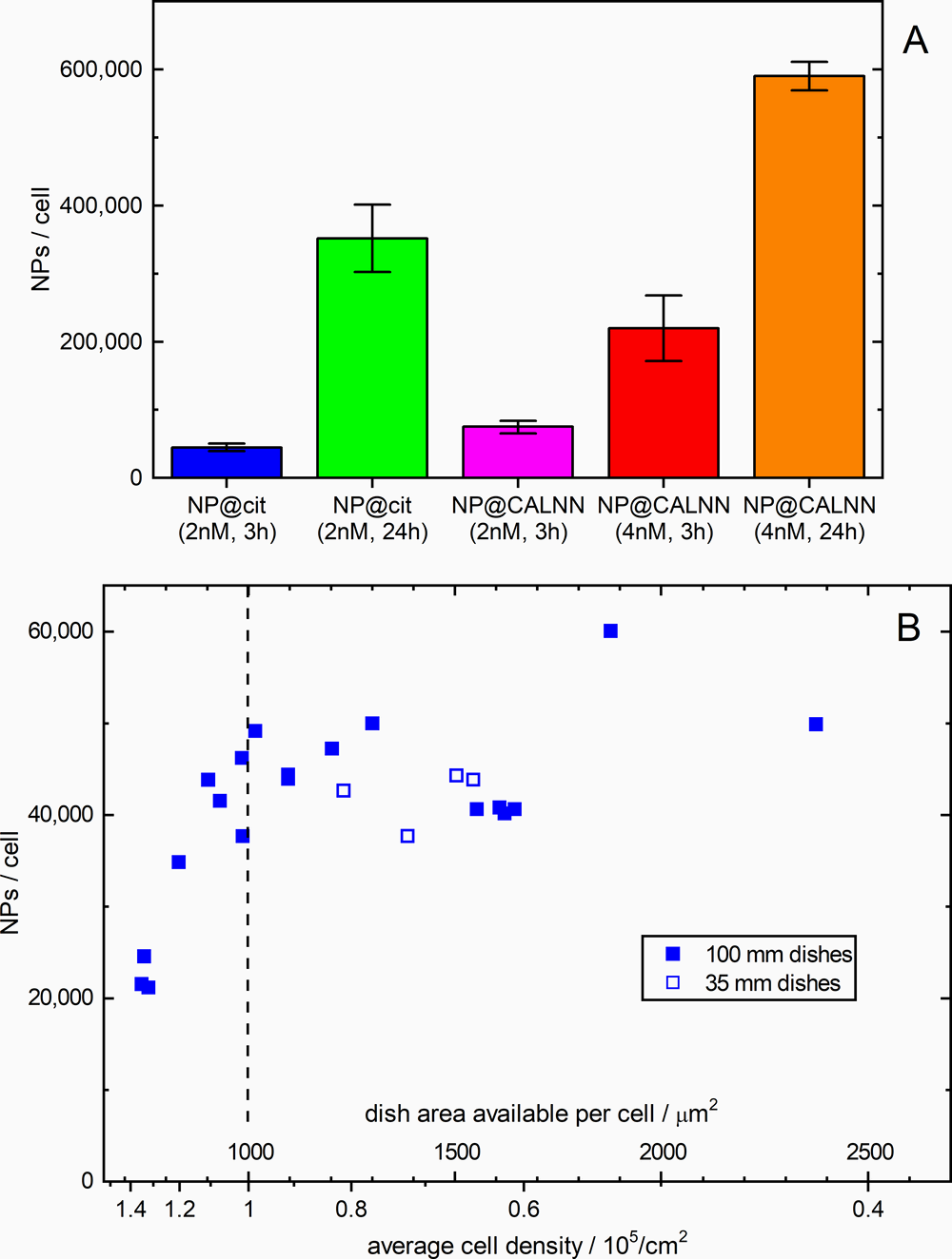
Association of 13–15 nm AuNPs with HeLa cells. (A) Average number of NPs/cell after incubation of cell cultures with a cell density of less than 10^5^ cells/cm^2^ with citrate- or CALNN-stabilized AuNPs at different concentrations for different incubation times; the error bars correspond to the standard deviation of individual experiments. (B) Results for individual cell culture dishes with a wide range of confluencies after incubation with 2 nM citrate-stabilized AuNPs for 3 h.

For this investigation, we used AuNPs with a core diameter of 13–15 nm, either as prepared, i.e., stabilized by a citrate capping layer, or modified with a covalently bound pentapeptide with the sequence CALNN. Capping AuNPs with CALNN has been shown to yield highly stable AuNPs even at high ionic strengths,^29^ and we were interested in the effect of this biomimetic capping layer on cell uptake and the outcome of laser irradiation experiments. The amount of citrate or CALNN-stabilized AuNPs that had become associated with HeLa cells during incubation for different times at different AuNP concentrations, determined using ICP-OES, is shown in Figure 1A; these values refer to the uptake under low confluency conditions, where the cells are free to grow to their maximum size; the numbers are summarized in Table S1 in the Supporting Information. The values for citrate-AuNPs (45,000 AuNPs/cell upon incubation at a AuNP concentration of 2 nM for 3 h) are in good agreement with values reported previously for HeLa cells incubated under similar conditions^30,31^ and indicate that uptake is proportional to the incubation time even for incubation for a full day. For CALNN-stabilized AuNPs, a larger amount of AuNPs was found to be associated with the cells, with the number being proportional to the AuNP concentration but saturating in less than a day.

These average numbers hide a significant cell-to-cell variation due to a variation of the individual cells’ surface area. In order to investigate this effect, the cell density on the culture dish was varied, which provides a simple method of reducing the average cell surface area exposed to the incubation solution.^32^ For cell cultures with low cell densities, cells are not size-restricted and were found to occupy on average an area of around 1500 μm^2^ on the dish, similar to the value reported for other epithelial cell lines.^32^ On the other hand, for essentially fully confluent cell cultures, the cells occupied on average an area of only 600 μm^2^; compare Section S3 of the Supporting Information. In these experiments, cells had been grown at most long enough to just reach confluency; significantly lower cell areas could be found upon prolonged cell culture growth. Thus, the extremes of our cell growth conditions (low cell density to just reaching confluency) lead to a variation of the average area occupied by an individual cell by a factor of 2.5.

At low confluency, the average area occupied by a cell decreases only slightly as the number of cells increases due to cell splitting (Figure S10) since new cells have enough space to move away from their parent cell.^32^ Therefore, not much change in AuNP binding is expected when varying the number of cells, and indeed, our AuNP uptake results show almost no variation of the number of citrate-stabilized AuNPs per cell at cell densities below 10^5^ cells/cm^2^; see Figure 1B. Upon reaching full confluence, above 10^5^ cells/cm^2^, the average cell size drops rapidly when further increasing the cell density (Figure S10), and Figure 1B shows that this is indeed accompanied by a significant decrease of the number of AuNPs per cell; for the highest cell density used here, the number of AuNPs per cell drops to less than half of the value observed for unrestricted cells, in good agreement with the variation of the (average) cell surface area. This suggests a close-to-linear relationship between the cell surface area and the number of citrate-stabilized AuNPs that bind to the cell, presumably due to the lower number of endocytosis receptors that are exposed to the incubation medium in smaller cells. It should be noted that this relationship only refers to the average AuNP binding of a cell population of identical size; single cell experiments have shown a significant variability of AuNP binding even for cells of the same size due to other effects, most likely a variability of endocytosis receptor density.^33,34^ As will be discussed in more detail below, this variability in AuNP binding and uptake presents difficulties in assessing the effectiveness of cytotoxic therapies mediated by AuNPs.

**Figure 2:**
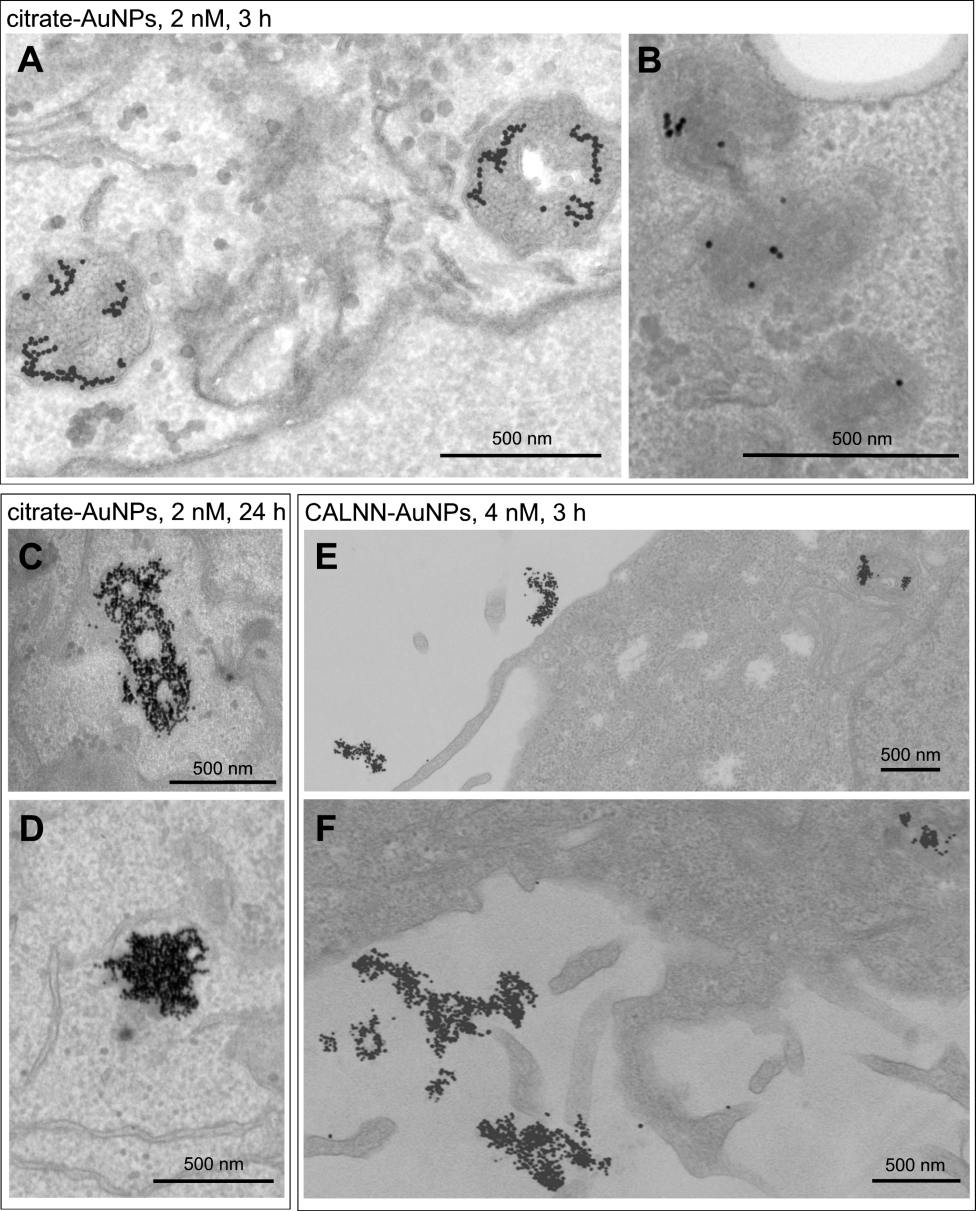
Representative TEM images of HeLa cells after incubation with 2 nM citrate-stabilized 13 nm AuNPs for 3 h (A, STEM; B, TEM) or 24 h (C + D, TEM) and with 4 nM CALNN-stabilized 13 nm AuNPs for 3 h (E + F, STEM).

We used electron microscopy to investigate the location of the AuNPs. TEM images of HeLa cells following incubation with 13 nm citrate-stabilized AuNPs show AuNPs in intracellular vesicles, including early and late endosomes and lysosomes (Figure 2), confirming endocytosis as the main mechanism for uptake.^23,30^ After incubation for 3 h, both individual and partly aggregated AuNPs were observed (Figure 2A,B), whereas incubation for 24 h results in extremely dense packing within the vesicles (Figure 2C,D). No significant binding of citrate-stabilized AuNPs to the cell surface was observed in these TEM images. In contrast, after incubation with CALNN-stabilized AuNPs, which leads to a larger number of AuNPs being associated with the cells as detected by ICP-OES, the majority of AuNPs are actually attached to the outside of the cell membrane rather than inside intracellular vesicles, as shown in Figure 2E,F. This shows that CALNN-stabilized AuNPs interact with HeLa cells by a different mechanism.

A semiquantitative analysis of the number of AuNPs in the TEM images provided estimated loadings of between 20,000 and 50,000 AuNPs per cell after incubation with citrate-stabilized AuNPs (2 nM, 3 h) and approximately 150,000 AuNPs per cell after incubation with CALNN-stabilized AuNPs (4 nM, 3 h), which is in good agreement with the ICP-OES results; see Section S2.2 of the Supporting Information. More importantly, this analysis suggests that only approximately 10% of the CALLN-stabilized AuNPs that are associated with cells after repeated washing with PBS are located inside the cells and the majority reside on the cell surface, whereas for citrate-stabilized AuNPs, more than 90% of the AuNPs are located inside the cell, in agreement with a previous report for other cell types, which used an analytical method rather than imaging for distinguishing between binding to the cell surface and uptake into the cell.^35^ The high AuNP density within the endocytic vesicles in cells incubated for 24 h made it impossible to estimate the AuNP loading for these cases.

UV–vis spectra of HeLa cell cultures after incubation with citrate- and CALNN-stabilized AuNPs for 3 h further confirmed the ICP-OES uptake results; see Section S2.3 of the Supporting Information.

### Cell Viability upon Nanoparticle Incubation

HeLa cells incubated with 13–15 nm AuNPs continue to split and proliferate at almost normal rates (compare Figure S11 in the Supporting Information), and application of trypan blue 24 h after exposure of the cells to AuNPs did not result in staining of cells, indicating that all cells had an intact cell membrane at this time. Quantitative analysis shows that the cell growth rate, i.e., the increase of the number of cells over 24 h, is slightly reduced after incubation with citrate-stabilized AuNPs for 3 h compared to cell dishes which were exposed to a 1:1 (v/v) mixture of CCM and Milli-Q water for the same time, from (2.05 ± 0.06) to (1.68 ± 0.19), in agreement with previous reports for the incubation of HeLa cells with citrate-stabilized AuNPs under similar conditions.^31,36^ Incubation with 4 nM CALNN-stabilized AuNPs for 3 h, on the other hand, did not lead to any measurable reduction of the growth rate (2.07 ± 0.08). All viability results reported below compare the cell growth rate of irradiated cells to non-irradiated cells on the same dish, which have experienced identical AuNP incubation conditions.

### Cell Viability upon Laser Irradiation

In the absence of AuNPs, irradiation of HeLa cells with cw laser light at 532 nm at the maximum intensity available (210 W/cm^2^) for up to 5 min does not significantly affect the cells. 24 h after irradiation, the trypan blue assay does not reveal any dead cells and the cells have a normal morphology; see Figure S12 in the Supporting Information. Moreover, the cells have undergone significant splitting, although a quantitative analysis shows that overall cell viability is reduced to 0.85 ± 0.08 for 3 min irradiation, compared to cells on the same dish that had not been irradiated, which showed the normal growth rate. Data for other irradiation conditions (Table S2 in the Supporting Information) show that decreasing the laser intensity reduces the effect of irradiation and that for the same cumulative dose the effect is larger for longer irradiation times at a lower dose. This is in agreement with literature reports that in vitro irradiation of various benign and malignant cell types with green or orange cw light has no or only minimal effects on their viability, even at intensities comparable to those used here.^1,37^

### Irradiation in the Presence of AuNPs

**Figure 3:**
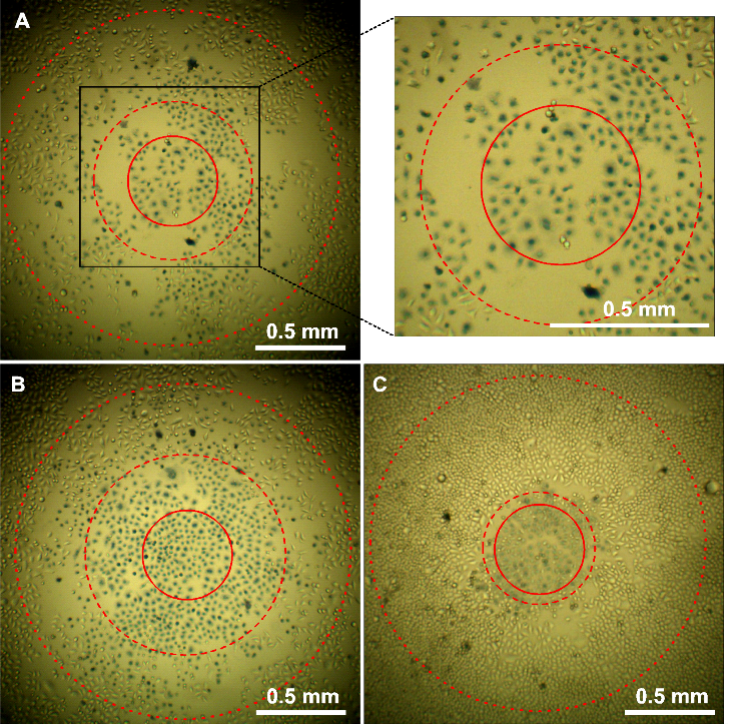
HeLa cells incubated with 13 nm citrate-stabilized AuNPs (2 nM, 24 h) prior to irradiation at 210 W/cm^2^ for 3 min (A, B) or incubated with 13 nm CALNN-stabilized AuNPs (4 nM, 3 h) prior to irradiation at 210 W/cm^2^ for 1 min (C). All images were taken after trypan blue treatment at 24 h after irradiation. The red solid circles indicate the center exposed area with a diameter of 0.5 mm, the dotted circles indicate the 1/e^2^ diameter of the laser beam, and the dashed circles indicate the approximate area in which all of the cells are dead. The image to the right of panel (A) is a magnified view of panel (A), as indicated.

**Figure 4:**
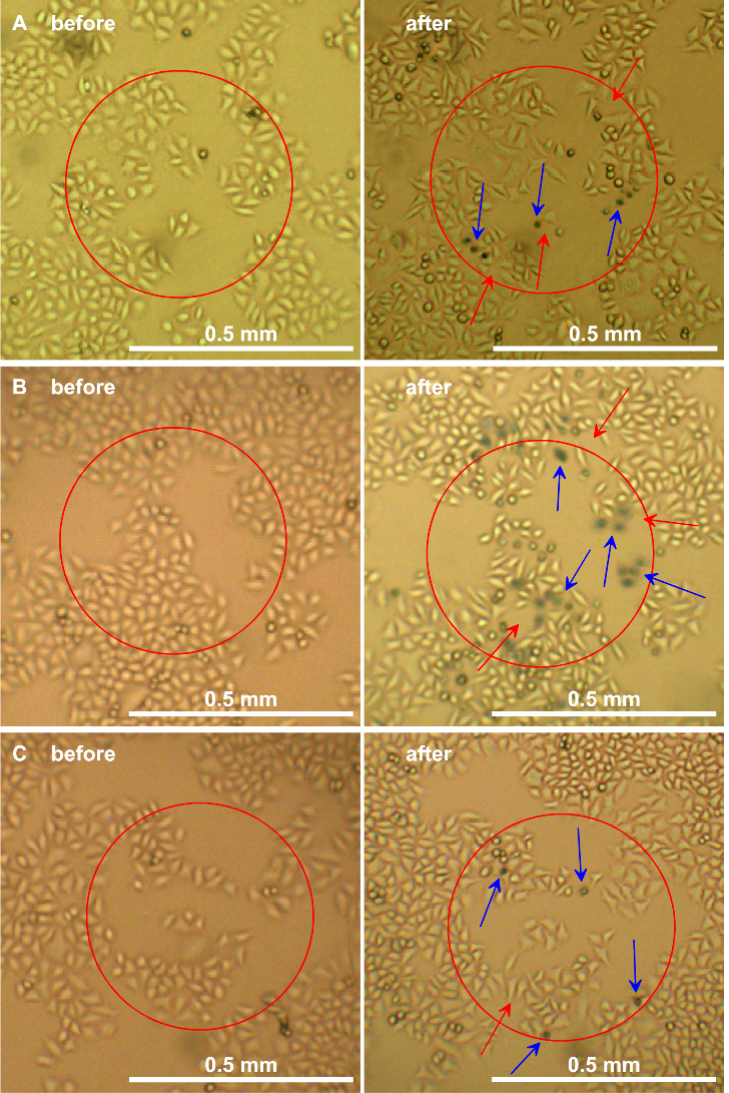
HeLa cells incubated with 13 nm citrate-stabilized AuNPs (2 nM, 3 h) (A) or incubated with 13 nm CALNN-stabilized AuNPs (4 nM, 3 h) (B, C) prior to irradiation at 140 W/cm^2^ for 3 min (A, B) or at 210 W/cm^2^ for 1 min (C). For all cases, the left image was taken just before irradiation and the right image after trypan blue treatment at 24 h after irradiation. The red solid circles indicate the center exposed area with a diameter of 0.5 mm used for cell counting. The blue arrows highlight some of the cells that have been stained by trypan blue, indicating that their cell membrane is compromised; the red arrows show where cells have detached from the dish after irradiation.

In contrast to the control experiments, for HeLa cells containing AuNPs, a measurable reduction of viability was observed upon irradiation with light at 532 nm, which overlaps with the plasmon resonance band of AuNPs (see Figure S1 in the Supporting Information). Two phenomenologically different outcomes were found when applying the trypan blue assay 24 h after irradiation, which are shown in Figures 3 and 4, respectively.

Figure 3 shows different situations where all cells were killed in a large area in the center of the laser beam, as clearly shown by their blue staining, although the size of the area varies and the area is surrounded by a transition zone where dead and live cells coexist. This result was often found upon irradiation at the highest laser intensity, but only after incubation of HeLa cells with citrate-stabilized AuNPs for 24 h (Figure 3A,B) or with CALNN-stabilized AuNPs for 3 h (Figure 3C), both of which result in the presence of a large number of AuNPs in the cell culture, either inside the cells (citrate-stabilized AuNPs) or mostly bound to the cell membrane (CALNN-stabilized AuNPs). In comparison, even at the highest laser intensity, this outcome was only very rarely observed after incubation with citrate-stabilized AuNPs for 3 h, which results in a higher uptake of AuNPs inside the cells than 3 h incubation with CALNN-stabilized AuNPs.

In contrast, Figure 4 shows experiments where irradiation has resulted in some dead cells whose cell membrane is defective so that they are stained by trypan blue incubation (blue arrows); in addition, some cells disappeared completely (red arrows), which for HeLa cellsnormally only happens upon cell death. On the other hand, interspersed with these cells are cells which have normal morphology and are not stained by trypan blue. Cell counting resulted in viabilities of 0.59, 0.33, and 0.77 for the experiments shown in Figure 4A–C, respectively, indicating the combined effect of cell death and reduced cell splitting during the 24 h after irradiation. Outside of the irradiated area, normal cell splitting was observed. This type of outcome was normally observed after incubation with citrate-stabilized AuNPs for 3 h, irrespective of the irradiation conditions, although cell viability did show a correlation with the irradiation conditions; see below. However, this outcome was also found after incubation with CALNN-stabilized AuNPs, particularly when using cell cultures with lower confluency.

The different outcomes shown in Figures 3 and 4 are partly caused by different AuNP loadings and irradiation conditions, but they cannot be ascribed solely to these factors. In many experiments, different outcomes were found for the same incubation and irradiation conditions; compare Figures 3C and 4C. In these cases, the only difference between the experiments resulting in complete cell killing and those resulting in a reduction of cell viability was the local cell confluency.

The well-defined circular area of complete cell killing which is shown in the examples of Figure 3 is reminiscent of results which have been reported previously for AuNP-induced PTT.^7,8,10^ This effect is based on the fact that when AuNPs absorb light, they dissipate virtually all of the absorbed light energy as heat to their surrounding on the picosecond time scale^18^ Given the cell dimensions and the thermal properties of biological material, the released heat then diffuses through the cell on the sub-millisecond time scale, thus rapidly heating the whole volume containing cells and its surrounding; in our case, this will mostly heat the buffer which the cells are immersed in, resulting in largely homogeneous heating of buffer and cells in the irradiated area.^23,38^

The effect of elevated temperatures on HeLa cell viability was investigated by immersion of culture dishes in a water bath. Incubation at 40 °C was found to not have any effect on cell viability, whereas keeping the cells at a temperature between 45 and 50 °C for 4 min in the absence of AuNPs resulted in a reduction of cell viability to ∼ 50%, and heating to above 50 °C for 4 min reduced the viability to 0–10%, in good agreement with the effects found for other cell lines.^3,4,39^ More details of these experiments are given in the Supporting Information, Section S5.

In order to quantitatively correlate our results with the temperature increase caused by the irradiation and thus confirm the cause of cell death as photothermal heating, we performed numerical simulations. For each individual irradiated area, the fraction of incident light which is absorbed by the AuNPs was calculated based on the local density of cells and the number of AuNPs taken up by each cell (Figure 1A). The explicit spatio-temporal temperature distribution in the dish during irradiation was calculated using finite-element dynamic heat transport simulations based on the Fourier equation.^23,38^ More details and example results are given in the Supporting Information, Section S6. It is worth noting that most of the temperature increase in the center of the laser beam is achieved in the first 30 s of irradiation, although the affected area grows with time.

**Figure 5:**
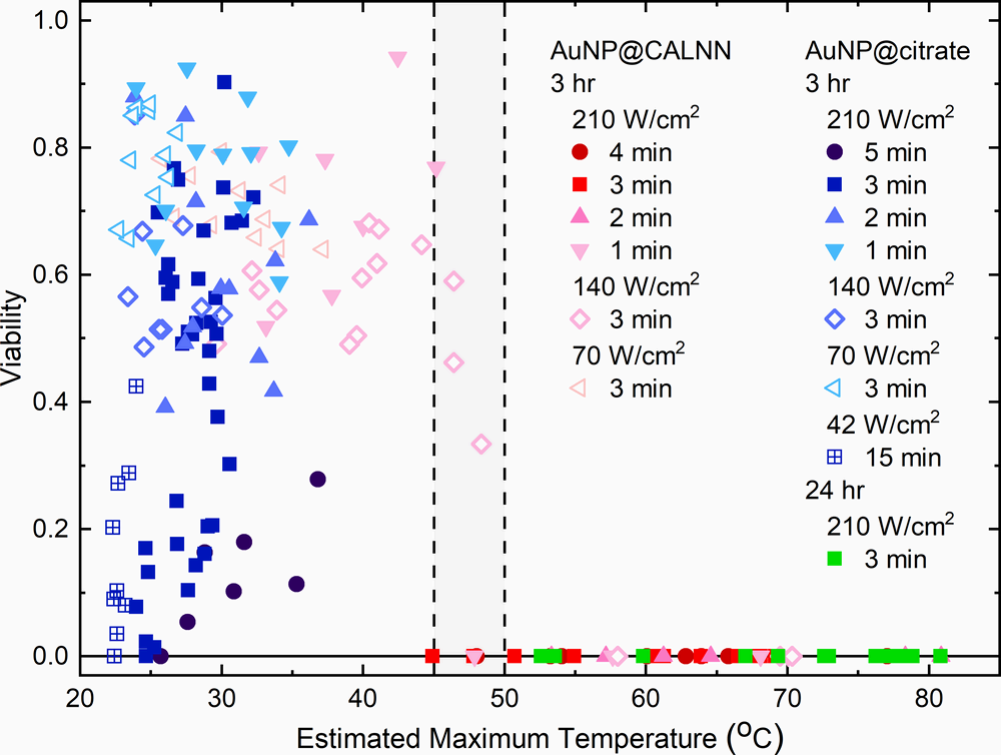
Viability of HeLa cells after incubation with 13–15 nm citrate-stabilized AuNPs (2 nM) or CALNN-stabilized AuNPs (4 nM) and irradiation at different laser intensities for different times. Shown here are the viabilities determined in individual experiments under the conditions indicated in the legend, plotted against the temperature in the center of the laser spot at the end of irradiation, and calculated for each individual experiment as described in the text. The gray area indicates the temperature range below which immersion in a water bath does not affect cell viability, whereas immersion above this range leads to essentially complete killing of cells.

Figure 5 correlates the observed viability and the calculated temperature in the maximum of the laser spot at the end of the irradiation for all experiments, which use different types of AuNP coatings, AuNP incubation times, and irradiation conditions. From these results, it is obvious that whenever the cells are heated to above 50 °C, they all die, whereas when the maximum temperature is below 45 °C, cell viability may be reduced, but in most cases, we did not observe complete cell killing. In a transition region between these two temperatures, both outcomes were found, although this might be largely due to the uncertainty of our temperature estimates. This observation is in very good agreement with the results obtained when using a water bath for heating the cell culture dish. Therefore, we conclude that under conditions where irradiation results in a temperature above 45–50 °C, all cells are killed by the photothermal effect. However, a significant reduction of cell viability, up to complete killing, was also found in experiments where our temperature estimates rule out significant heating. This effect must arise from a photochemical effect, and we suggest this to be the photogeneration of singlet oxygen (^1^O_2_) by AuNPs,^14,19−22,24,25^ which is generally believed to be the main ROS species involved in PDT. In the following, we will discuss these two different mechanisms in more detail.

### Photothermal Cell Killing

There have been many reports of in vitro AuNP-induced PTT,^1−14^ although in most cases there was no attempt to determine the temperature achieved during irradiation and thus verify the cell killing mechanism to be PTT, as opposed to AuNP-induced PDT. Only in a few reports was the rise of the cell culture temperature measured explicitly.^9,11,12^

Our current results confirm that AuNP-induced PTT does kill HeLa cells when the local temperature reaches values above 50 °C. For almost all experiments which result in complete cell killing in the center of the irradiated area, such as the ones shown in Figure 3, the temperature reached a value above 50 °C throughout the affected area, and conversely, whenever this temperature is reached, all cells die (Figure 5). The size of the central area with complete cell death is larger when more laser light is absorbed, either because of stronger or longer light irradiation or because of the presence of more AuNPs, irrespective of their location (on the cell surface or in the cell interior). This shows that complete cell death normally is limited to those parts of the cell culture where a local temperature of 50 °C or above is reached. In many cases, this central area of complete cell death is surrounded by a transition zone of decreased cell viability, where some cells are killed but others survive, similar to the outcome seen throughout the irradiated area for those experiments resulting in a maximum temperature increase to less than 45 °C, as expected.

Complete cell killing was often found after incubation with citrate-stabilized AuNPs for 24 h or with CALNN-stabilized AuNPs for 3 h, both of which result in large AuNP uptake (Figure 1), albeit in different locations (Figure 2). In contrast, for HeLa cells incubated with citrate-stabilized AuNPs for only 3 h, even irradiation at the highest laser power for up to 5 min did not result in a temperature increase above 35 °C, and only in a few of these cases was a situation of zero viability (all cells killed) found. Interestingly, both outcomes (all cells killed in the central area vs reduced viability only) were found for some conditions, such as incubation with CALNN-stabilized AuNPs for 3 h and irradiation with 210 W/cm^2^ for 1 min (compare Figures 3C and 4C) or 140 W/cm^2^ for 3 min, with the outcome determined by the local cell confluency in the irradiated area, which directly affects the heat deposited and thus the temperature increase in the laser spot; compare Figure 5.

Thus, we can conclude that under our experimental conditions, using cw irradiation of a relatively large area, we can kill HeLa cells by AuNP-induced PTT, particularly when high AuNP uptake into the cell culture is achieved. This effect is independent of the location of the AuNPs, which may be internalized (citrate-stabilized AuNPs) or mostly attached to the cell membrane (CALNN-stabilized AuNPs). Most importantly, the cell-to-cell variation of AuNP uptake does not affect the outcome. This is because rapid heat diffusion leads to largely homogeneous heating within the laser spot, which is much larger than the cell size, so that all cells are killed within the affected area, independent of their individual AuNP content, in contrast to the photochemical effect; see below. Using typical heat diffusion parameters for biological material^40^ and typical cell dimensions (10 μm), it can be estimated that any heat deposited inside a cell or at its surface will equilibrate over the whole cell volume on the sub-millisecond time scale. Given the time scale of our experiments, this means that no significant localized heating on the subcellular or cellular length scale can be achieved. This is very different from irradiation using short laser pulses or strongly focused laser beams; short laser pulses deposit heat in such a short time that it cannot diffuse away during deposition, thus potentially leading to extremely high localized temperatures, albeit only for short times, whereas a strongly focused laser beam can have an intensity which is many orders of magnitude higher than the intensities used here, thus achieving very high temperatures, albeit only on subcellular length scales. In both cases, the biological reaction of the cell might be very different from standard thermal therapy.^4^ However, irradiation with short laser pulses requires more sophisticated laser systems, whereas focused irradiation requires well-controlled scanning of the laser spot over the treatment area. Hence, in a clinical setting these approaches would add complexity to the protocol and might not be useful for practical applications.

Although no explicit test for the mechanism of cell death was performed here, we note that under conditions where complete cell death was achieved by inducing high temperatures, compromised cell membranes often were observed immediately after irradiation, see Figure S16 in the Supporting Information, which shows that immediately after laser irradiation cells in the center of the laser beam were stained by trypan blue. This suggests that AuNP-induced PTT acts via necrosis rather than programmed cell death (apoptosis), as expected for heating above 50 °C,^4^ although the possibility of apoptosis induced by heating to 45–50 °C has also been reported.^13^

### Photodynamic Effects

Very different results were obtained when performing experiments under conditions resulting in heating to less than 45 °C; see Figure 4. Images obtained under these conditions often show no well-defined area where all cells are dead but show live and dead cells in close vicinity and interspersed throughout the irradiated area. In some cases, we even found evidence of continued cell splitting in the center of the irradiated area; see Figure S3 in the Supporting Information. Unlike the almost instantaneous cell death upon heating to 50 °C or above, compromised plasma membranes are only detectable hours after irradiation; see Figure S17 in the Supporting Information. This is more suggestive of secondary necrosis following apoptosis as the mechanism for cell death, but again, we did not explicitly investigate this question here. Moreover, we noted that increasing the cell confluency, which will result in more AuNPs in the irradiated area and hence more heating upon irradiation, seems to reduce the extent of cell death and increase viability, as long as the final temperature remains below 45 °C.

These phenomenological differences suggest that a different underlying effect is responsible for cell killing. In particular, the effect is confined to individual cells, which is impossible when relying on the photothermal route, where larger areas of the cell culture are affected, because of the unavoidable heat diffusion. We suggest this effect to be the photogeneration of singlet oxygen by AuNPs,^14,19−22,24,25^ which has been suggested previously to result in cell killing, both in vitro and in vivo,^14,19−24^ although in many cases no clear evidence was provided to rule out cell killing by the PTT effect. The lifetime of singlet oxygen in aqueous solution or the cell environment is on the order of a few microseconds, resulting in a diffusion-limited range of activity of at most a few 100 nm,^41^ which is significantly shorter than a cell’s dimension and therefore limits its effects to the cell in which it has been generated.

Our results also provide evidence that singlet oxygen must be generated inside the cell to have an effect on cell viability. Figure 5 shows that under the same irradiation conditions, cells incubated with CALNN-stabilized AuNPs tend to be heated significantly more than cells incubated with citrate-stabilized AuNPs for the same time due to the significantly larger number of CALLN-stabilized AuNPs that are associated with cells. This often results in heating above 50 °C and hence complete cell death. However, if the temperature remains below 45 °C, CALLN-stabilized AuNPs do not have a larger effect on viability than citrate-stabilized AuNPs under the same irradiation conditions, in spite of the larger number of AuNPs present. This can be ascribed to the fact that, although the total number of CALNN-stabilized AuNPs associated with cells is much larger, only a small fraction is taken up into the cell, see above, so overall a similar number of CALNN- and citrate-stabilized AuNPs are inside the cell. This suggests that even at the highest irradiation intensities not enough singlet oxygen is produced by CALNN-stabilized AuNPs bound to the outside of the plasma membrane to disrupt the membrane, which would result in rapid necrosis, as has been observed with standard PDT sensitizers that predominantly bind to the membrane,^26^ and only the internalized AuNPs result in the PDT effect.

### Dependence of Photodynamic Effect on Cell Size

**Figure 6:**
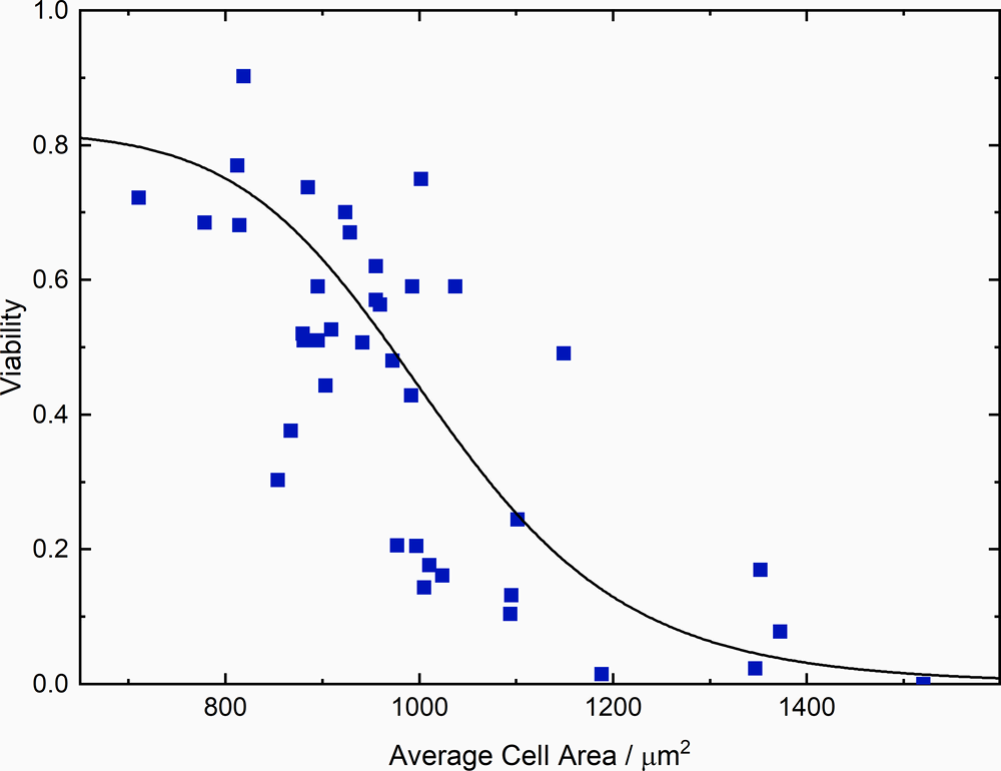
Viability of HeLa cells after incubation with 13 nm citrate-stabilized AuNPs for 3 h and irradiation at 210 W/cm^2^ for 3 min. The viability data (as measured in individual experiments) are plotted against the average cell area for each individual experiment. The solid line is a guide for the eye.

Figure 5 clearly shows that cell viability upon laser irradiation is highly variable even when comparing results obtained under identical experimental conditions. This variability is most pronounced when irradiating cells with 210 W/cm^2^ for 3 min after incubation with citrate-stabilized AuNPs for 3 h, where viabilities in the range of 0–0.9 were found, the latter value corresponding to the value observed for this irradiation in the absence of AuNPs. It seems that these experiments result in conditions which are close to the threshold for killing a cell and therefore show the highest variation in viability. We found that a significant contribution to this variability is the variation of local cell density between different irradiated areas, which results in a variation of the cell surface exposed to the culture medium and hence the number of AuNPs taken up by an individual cell, as discussed above. This is clearly shown in Figure 6, where cell viability is shown against the average size of the irradiated cells, measured for each of the irradiated areas as described in Section S3 of the Supporting Information. This shows a clear trend—as the average cell size increases, the PDT efficacy increases; thus, cells that are less restricted by high local confluence have lower viability under otherwise identical conditions, presumably because they take up more AuNPs, resulting in a higher dose of singlet oxygen upon irradiation. The figure also shows that under these incubation and irradiation conditions, unrestricted cells, which have a cell area of up to 1500 μm^2^, are almost completely killed.

Even after accounting for the average cell size, a significant scatter of the observed cell viability remains, particularly for conditions of intermediate local cell density. We primarily attribute this to the observation that under these conditions there are limited numbers of restricted and unrestricted cells in the irradiated areas; see Figure 4. This leads to a significant variation of the distribution of cell sizes between different experiments, even when considering only those with a similar average cell area. Since cell death upon irradiation occurs when the cell has a certain size and therefore contains more than a threshold number of AuNPs, this leads to a scatter of the viability—different size distributions with the same average cell area have different fractions of cells containing at least that threshold number of AuNPs.

Some other effects may further increase the scatter of cell viability under identical conditions. (i) Both individual and partially aggregated AuNPs are found in intracellular vesicles (Figure 2), and the latter have been shown to be less efficient at rupturing the vesicles,^23^ possibly due to restricted access of O_2_ to the AuNPs or due to rapid quenching of photoinduced singlet oxygen. The random distribution of individual and aggregated AuNPs will increase the variability of the resulting effects. (ii) The spatial distribution of AuNP-containing endosomes or lysosomes varies from cell to cell. Due to the short lifetime of singlet oxygen and its resulting short diffusion distance, this may contribute significantly to the viability variation since the susceptibility of different cell organelles to singlet oxygen triggering cell death varies significantly.^26^ (iii) Most cellular processes show significant cell-to-cell variability, which may be related to the phenotypic state or population context of individual cells, independent of cell size.^42^ In particular, the density of receptors on cell surfaces has been shown to have significant cell-to-cell variability, which leads to significant variability of nanoparticle uptake even when corrected for cell size.^33,34^

### Dose Dependence of Photodynamic Effect

**Figure 7:**
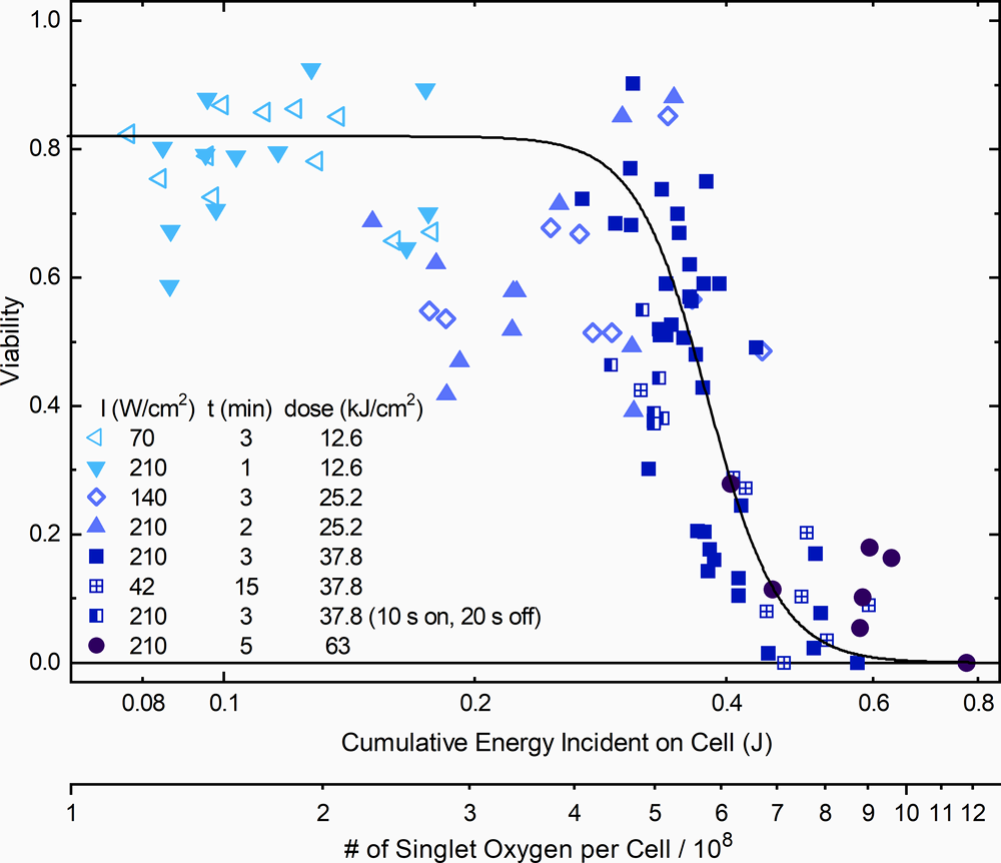
Viability of HeLa cells (as measured in individual experiments) after incubation with 13 nm citrate-stabilized AuNPs (2 nM) for 3 h and irradiation at different laser intensities and irradiation times, plotted against the cumulative light energy to which the average cell was exposed in the experiment, calculated from the product of the average cell area, laser intensity, and irradiation time. The second *x*-axis provides a rough estimate of the amount of singlet oxygen produced during irradiation, as described in the text. The solid line is a guide for the eye.

An increase of the total irradiation light dose, by increasing the laser intensity or irradiation time, leads to a reduction of the viability of HeLa cells since a larger light dose will create more ROS, most likely singlet oxygen. However, in Figure 5, the effect is partially obscured by the variability arising from the variation of local cell confluence discussed above. In Figure 7, this variation is accounted for by plotting the viability against the product of the average cell area and the cumulative light dose (intensity × time); this yields a measure of the total amount of light energy incident on each cell. In turn, this can be used to estimate the amount of singlet oxygen produced by the AuNPs during the irradiation, assuming that the number of AuNPs taken up increases proportional to the cell surface area, as suggested by our detailed AuNP uptake analysis. This figure shows clearly that cells exposed to a low cumulative light dose are not greatly affected by the irradiation, and that above a certain threshold essentially all cells are killed, with a relatively broad transition region arising from the effects discussed above.

Figure 7 shows more clearly that the photochemical effect only depends on the cumulative light dose on each cell—the same results are obtained upon irradiation at 210 W/cm^2^ for 3 min and at 42 W/cm^2^ for 15 min, and it makes no difference if the total dose is delivered in one single irradiation event or in short bursts of 10 s duration, interrupted by 20 s intervals of darkness, as long as the total light dose is the same; the same is true for other cumulative doses. This result is in agreement with previous observations for PDT-induced in vitro cell killing.^43−45^ It should be noted that very different temperature profiles result from these different irradiation modalities since the time scale of heat diffusion out of the irradiated area is shorter than the experimental time scales, which again rules out that cell death is caused by thermal effects and provides further confirmation of our assignment of PDT as the relevant mechanism under these conditions.

It appears that for these incubation conditions (13 nm citrate-stabilized AuNPs at 2 nM for 3 h), exposure of a cell to a cumulative light dose of around 0.4 J at 532 nm defines a threshold, below which only a small fraction of cells are affected by irradiation, whereas above it, a significant fraction is killed, which can reach up to 100% for the larger cells found under low local confluency conditions. It should be noted that the AuNPs only absorb ∼0.3% of this incident light, as can be estimated from the AuNP uptake data (∼ 50,000 AuNPs/cell for the largest cells with an area of 1500 μm^2^) and the extinction coefficient of AuNPs.^46^Section S8 of the Supporting Information describes experiments using the MTT assay, which result in essentially the same threshold cumulative incident energy for cell killing as the more extensive results described here based on the trypan blue essay.

The quantum yield of photogeneration of ^1^O_2_ by citrate-stabilized AuNPs has been estimated to be on the order of 2 × 10^–7^ in air-saturated aqueous solution.^47^ Using this value and the fraction of incident light absorbed by AuNPs, the cumulative light dose incident on one cell can be converted to the cumulative number of singlet oxygen molecules generated; those numbers are included in Figure 7. This conversion should be regarded as a rough estimate, not least because the quantum yield of photogeneration of ^1^O_2_ depends on oxygen concentration and cell monolayers may become hypoxic during PDT treatment; furthermore, the dense packing of AuNPs in endosomes may interfere with the formation of ^1^O_2_. The threshold dose of 0.4 J corresponds to an amount of ∼6 × 10^8^ singlet oxygen generated in each cell or a cumulative ^1^O_2_ concentration of ∼0.3 mM, assuming a typical HeLa cell volume of 3000 μm^3^. This is comparable to the cumulative ^1^O_2_ concentration required for successful in vitro PDT treatment using standard molecular sensitizers or direct photoexcitation, which range from 0.2 to 5 mM for a range of different cell lines.^43−45^ This provides further evidence for PDT as the main mechanism of cell damage for our experiments, which do not lead to high-enough temperatures for PTT, although the exact threshold dose, as well as AuNP uptake, may to some extent depend on the cell type used or targeted in practical PDT applications.

It should be noted that the irradiation intensities used in our in vitro studies are too high for practical therapy applications. To overcome this problem, lower intensities could be used in combination with higher AuNP uptake, e.g., by incubation at higher AuNP concentrations and/or for longer times; targeted AuNP uptake (see below) will also result in higher uptake than the unspecific uptake used here. However, the observation that the PDT effect depends on the cumulative light dose also suggests that lower irradiation intensities could simply be compensated for by longer irradiation times. A combination of these approaches would easily bring the intensities to values that are acceptable for in vivo applications.

### Photodynamic vs Photothermal Therapy

Our results show clearly that AuNPs can trigger photoinduced cell death in vitro by two different mechanisms, PDT or PTT, i.e., the photogeneration of singlet oxygen or local heating upon irradiation. Which of these mechanisms prevails depends largely on the amount of AuNPs present, which can be adjusted by the AuNP capping layer, the incubation conditions, and the confluency of the cell culture. Obviously, both heat and singlet oxygen are generated under all conditions, but heat can only lead to cell death when local temperatures exceed 45–50 °C, whereas singlet oxygen is cytotoxic at all temperatures but requires the generation of a minimum cumulative amount of ∼ 6 × 10^8^ singlet oxygen molecules inside a cell. In our experiments, it was easy to heat the cells to 50 °C or higher upon incubation with citrate-stabilized AuNPs for 24 h or with CALNN-stabilized AuNPs for 3 h; for the former, most AuNPs were found in cytosolic endosomes, whereas for the latter, most AuNPs were attached to the cell surface, but this made no distinction for the photothermal effect, which can be easily rationalized by fast heat diffusion. Upon sufficient heating, all cells in the affected area were found to die. On the other hand, under conditions where fewer AuNPs were present, either because of shorter incubation periods or lower cell culture confluency, the temperature generally remained below 45 °C and irradiation often resulted in the coexistence of live and dead cells, showing the localized nature of the PDT effect, which results from the short lifetime and hence low range of activity of singlet oxygen.

These results show that it is important to carefully discriminate between PTT and PDT effects when investigating AuNPs as photoagents. Previous reports of in vitro cell killing by irradiation of AuNP-incubated cancer cells often ascribe the effect to PTT without clear proof that the cells actually had been heated to high-enough temperatures and thus ignore the PDT potential of AuNPs.^1−8,10,13,14^ This includes reports where the irradiation effect was independent of the initial temperature of the cells,^14^ reports of significant cell death upon heating to not much more than 40 °C for less than a minute,^13^ and experiments where a simple estimate shows that all of the incident light would need to be absorbed in a single cell layer and converted to heat,^28^ which is impossible given fundamental optical considerations. Our results suggest that in those cases, cell death may actually have been caused by PDT effects and not by the accompanying temperature increase. Only a few reports indeed show that killing of cells upon irradiation in the presence of AuNPs was accompanied by heating to above 45–50 °C.^9,11,12^

On the other hand, other in vitro studies claim to show AuNP-mediated PDT but offer no proof that there was no heating to relevant temperatures.^19,20^ In this context, it is highly important to consider not only the detailed experimental conditions, including the amount and distribution of AuNPs present, but also the physical mechanism of heat flow; for example, for long-enough irradiation times, a steady state will be reached where the heat generated and the outward heat flow equilibrate, so that the temperature in the heated volume does not increase any more (see Figure S15); under these conditions, the maximum temperature increase will be directly proportional to the power absorbed but is largely independent of the length of irradiation. Again, there are some exceptions, where the temperature was estimated from simulations similar to those used here,^23^ or other more indirect evidence for PDT as the prevailing mechanism is presented.^14,21,22^

A clear distinction between cell death by AuNP-induced PTT or PDT effects will also be important for investigating the cellular mechanism(s) that lead to cell death. It is generally believed that heating above 50 °C leads to instantaneous cell death via necrosis,^4^ whereas our results suggest that PDT effects at lower temperatures trigger programmed cell death (apoptosis), although we did not investigate this question in detail. Any future investigation of such effects will have to make sure that the therapy modality is clearly identified.

PDT has the clear advantage of having a highly localized effect due to the short lifetime and thus limited range of activity of singlet oxygen (∼100 nm).^41^ Therefore, preferential AuNP uptake by cancer cells, e.g., using suitable targeting ligands as described below, will allow selective cancer cell killing without direct detrimental effects on neighboring healthy cells. PTT, on the other hand, is not able to achieve such selective cell killing even when using targeted AuNPs since heat diffusion is too fast; using the typical thermal parameters of tissue,^40^ one can show that any heat deposited at one location will have spread over a range of 5 mm after only 1 min, which rules out the possibility of localized heating of individual cells or even microtumors under cw irradiation conditions.^23,38^ This is clearly visualized by the two phenomenologically different outcomes of the trypan blue cell killing assay after irradiation under PTT or PDT conditions, Figures 3 and 4, respectively, although it has to be noted that PDT is able to kill all targeted cells provided that enough AuNPs have been internalized and a sufficient cumulative light dose has been applied. In principle, cell-localized heating is achievable with highly focused cw light or short laser pulses,^38^ but both approaches result in significant challenges for practical applications. Furthermore, short laser pulses, which lead to nanoscale bubbles affecting cell structures by mechanical effects,^48^ also lead to AuNP reshaping^49^ and even fragmentation^50^ with unpredictable consequences, such as a change of their optical properties^49^ or increased cytotoxicity upon their release from the targeted cell.^51^

Thus, PTT with AuNPs may be a very useful technique for the treatment of larger solid tumors, although determination of the correct light dose to ensure that cancer cells at the tumor edge are reliably destroyed without significant damage to peripheral healthy cells will be difficult. Reliable killing of cancer cells in the tumor margin is particularly important in light of the suggestion that sublethal heating may increase the resistance of tumor cells to subsequent treatment due to the induction of heat shock protein expression.^39^ Targeting the AuNPs to cancer cells will reduce the effect on neighboring tissue, as well as the side effects of increased light sensitivity. In comparison, PDT with targeted AuNPs does not suffer from these problems due to the localized activity of singlet oxygen. Moreover, selective PDT with targeted AuNPs could also be used for the treatment of ill-defined micro-sized tumors, small metastatic tumors, or residual cancer cells after removal of the main tumor. The latter application would be particularly useful where the vicinity of vital regions or blood vessels does not allow for large surgical margins, e.g., in head-and-neck cancers, which results in an increased risk of tumor recurrence due to microscopic cancer tissue remaining in the tumor bed; currently, this is addressed by postoperative chemo- and/or radiotherapy, often causing significant long-term side effects.^15^ PTT would not be applicable in any of those cases because of its lack of selectivity at a cell-by-cell level. Furthermore, it has been observed that PDT may cause less damage to the extracellular matrix than PTT, which provides improved conditions for healing.^52^

### Advantages of AuNP-mediated PDT

Most conventional PDT-PSs are organic dye molecules and suffer from several drawbacks, including photobleaching, lack of stability against enzymatic degradation, limited targeting capability, and absorbance bands which often are located outside the “biological window” in the near-IR spectral region for which tissue has high light penetration.^4,24^ AuNPs are able to overcome all of these disadvantages and, although the quantum yield for ^1^O_2_ photogeneration by AuNPs is several orders of magnitude smaller than that of conventional PSs,^47^ their extinction coefficient is 4–5 orders of magnitude larger than that of organic dye molecules,^46^ which counteracts this low ^1^O_2_ efficiency.

Photobleaching describes the fact that the PS can be modified upon irradiation, for example, by a reaction with the ^1^O_2_ which has been generated by the molecule itself.^53^ This leads to a loss of absorbance and hence limits the number of excitation cycles and thus ^1^O_2_ molecules which each PS molecule can generate. AuNPs, on the other hand, are stable under irradiation and do not experience bleaching. Similarly, organic dyes, but not AuNPs, are prone to degradation by enzymes.^54^

Conventional PSs typically show some preferential accumulation in tumor tissues and cancer cells due to two passive targeting effects: (i) the enhanced permeability and retention (EPR), which arises from the leaky vasculature and poor lymphatic drainage of typical tumor tissues;^55,56^ and (ii) the hydrophobic nature of conventional PSs (arising from their extended conjugated π-bond systems), which leads to strong interaction with low-density lipoproteins, which are often overexpressed in cancer cells.^57^ However, neither of these effects is highly specific to cancer tissue,^58^ and therefore, PSs are distributed throughout the body; in particular, they are found in the skin, which causes one of the main side effects of PDT—prolonged light sensitivity. This systemic distribution of PS may also lead to damage to healthy tissue near the tumor site during treatment. Larger objects, e.g., AuNPs, have a significantly increased EPR effect, i.e., they show an increased differentiation between healthy and cancerous tissue,^55,56^ thus conferring an intrinsic advantage to AuNPs as novel PSs.

However, none of these effects are useful in the context of noncancer therapeutic PDT applications, such as antimicrobial inactivation.^27^ The EPR effect is also not of relevance for applications such as the treatment of ill-defined micro-sized tumors, small metastatic tumors, or the postsurgical removal of residual cancer cells in the tumor bed. More selective therapy results can be achieved by active targeting, i.e., coupling the PS to a ligand with high selective binding affinity to specific markers that are overexpressed on the targeted cells.^59,60^ Ligands that have been used for this purpose include antibodies and other proteins, peptides, folate, or saccharides.^55^ However, the design, synthesis, and characterization of organic dye–ligand conjugates require significant efforts, and different strategies are needed for each combination of PS and targeting agent, resulting in low flexibility of therapy applications. In contrast, due to their high affinity for binding to amine and thiol groups, AuNP surfaces can be easily modified and many well-characterized routes exist for the binding of different types of ligands,^61,62^ which allows for the rapid modification and adaptation to different targeting requirements. Such labeled AuNPs benefit not only from the enhanced EPR effect for nanosized objects but also from the selectivity toward the target cells, which is highly increased by the targeting ligand. This approach has been tested in the context of AuNP-mediated PTT^1,2,6,28^ but applies similarly to PDT.

For most PDT applications, the PS should ideally have a strong absorbance in the “biological window” above 700 nm; there are some exceptions, such as some skin cancers or the postsurgical removal of residual cancer cells in the tumor bed. Although the spherical AuNPs used here have their surface plasmon band at 520 nm, and therefore will find limited application for in vivo studies or practical applications, the surface plasmon band can be shifted into the biological window by using AuNPs with different shapes, such as shells, rods, or stars, thus overcoming the difficulty of synthesizing organic molecules with strong absorbance in the near-IR. AuNPs with such shapes have been confirmed to generate singlet oxygen upon irradiation, often with increased singlet oxygen quantum yields.^14,19−22,24^

It is also of interest to consider the cellular mechanism of cell death induced by AuNP-mediated PDT. Many conventional PSs target organelles, such as mitochondria or the endoplasmic reticulum, to trigger apoptosis signaling pathways. For this, they need to be located at or in those organelles due to the short lifetime of singlet oxygen, which limits its range of activity to ∼100 nm.^41^ Endocytosed AuNPs, on the other hand, are initially located exclusively within endosomes (see Figure 2), which eventually fuse with lysosomes. Upon light irradiation, AuNPs cause permeabilization and destruction of these vesicles by the ROS generated and are then free to diffuse through the cytosol.^23^ Although diffusion in the crowded environment of the cytosol is complex and characterized by a size-dependent viscosity,^63^ AuNPs with 15 nm diameter can diffuse over the full cell volume within a few minutes, thus potentially reaching the same organelles during our irradiation intervals and triggering the same signaling pathways as conventional PSs. However, the lysosomal membrane rupture itself releases cathepsins, which can trigger apoptosis;^64,65^ thus, it may not be necessary for AuNPs to translocate to specific organelles. Lysosome photodamage has also been shown to enhance the effects of mitochondrial photodamage^66^ and has even been suggested to result in cell death by autophagy rather than apoptosis.^67^ In this context, it is of interest that in many cancer cells lysosomal cathepsins are overexpressed,^64,65^ which will result in enhanced PDT effects on cancer cells compared to normal cells even in the absence of specific targeting. On the other hand, apoptosis in general is downregulated in cancer cells,^65,68^ so that lysosome-targeted PDT, which increases the apoptosis signal or even triggers autophagy programmed cell death by lysosome disruption, might be a more potent approach for PDT cancer treatment than targeting mitochondria. Both effects would give AuNPs an intrinsic advantage over more conventional PSs.

Another advantage of the use of AuNPs is the fact that upon irradiation most of the light energy which is absorbed is converted to heat.^18^ Therefore, AuNPs can be used as PTT agents by heating the tissue to temperatures above 45–50 °C, as described above. However, even in situations where one wants to avoid heating to such temperatures to avoid large-scale indiscriminate cell killing, gentle heating to temperatures which on their own can be tolerated by cells can result in higher susceptibility of the cells to PDT treatment,^69^ most likely due to an Arrhenius-type acceleration of the photosensitization reactions.^70^ In vivo, additional effects of a slightly raised temperature will be beneficial for PDT, such as the increased blood flow and the shift of the hemoglobin–oxygen equilibrium, which improve the supply of oxygen.^71^

The PDT effect of AuNPs can be further enhanced using traditional PSs as ligands,^6,24^ in which case improved ROS generation may be achieved by the plasmon field enhancement effect, although a more important function is the ability to improve targeting using AuNP functionalization with multiple ligands. On the other hand, photobleaching and enzymatic degradation drawbacks remain when using this approach. AuNPs are also efficient radiosensitizers for radiotherapy and can transport chemotherapy agents into cancer cells. They can act as contrast agents for X-ray CT-imaging or carry fluorescence or Raman spectroscopic markers, and their rapid heat transduction makes them useful for photoacoustic imaging. Thus, AuNPs also offer the possibility of multimodal therapy or theranostic approaches.^4,6^

## Conclusions

AuNPs internalized by HeLa cells can cause cell death upon irradiation with visible light via two significantly different pathways: photothermal heating or photogeneration of singlet oxygen, i.e., a photodynamic effect. These two treatment modalities occur in parallel, which often has been ignored in previous work, although they can lead to significantly different outcomes. Which modality dominates depends critically on experimental details, in particular, the amount and location of the AuNPs. The photothermal effect is independent of the location of the AuNPs and affects all cells within the irradiated volume, which can be rationalized in terms of the fast diffusion of heat. In contrast, the photodynamic effect requires the AuNPs to be internalized, which typically happens via endocytosis, and only cells containing a sufficient number of internalized AuNPs will be affected. This shows that AuNPs are potential PDT agents which could provide advantages over conventional organic PSs, such as high photostability and absence of enzymatic degradation. Most importantly, the combination of a highly localized effect with the ability to selectively target different cell types using suitable AuNP ligands will allow for treatment modalities where only the target cells, e.g., cancer cells, are affected, with only minor direct effects on neighboring healthy cells, which is not possible when using a photothermal approach.

## Data Availability

The data underlying this study are openly available from the University of Liverpool Research Data Catalogue at 10.17638/datacat.liverpool.ac.uk/2483.

## Supporting Information Available

Experimental details; AuNP uptake quantification using ICP-OES, electron microscopy, or UV–vis spectroscopy; investigation of HeLa cell sizes at different confluencies; control experiments (HeLa cell viability upon AuNP incubation in the absence of irradiation or upon irradiation in the absence of AuNPs); effect of elevated temperatures on HeLa cells; finite-element simulations of temperature profile upon laser irradiation of cell cultures; time course of HeLa cell death after irradiation in the presence of AuNPs; and investigation of HeLa cell killing upon irradiation in the presence of AuNPs using the MTT assay (PDF)
