# Supplementary material for: Gold Nanoparticles for Photothermal and Photodynamic Therapy
Source: ACS Omega. 2024 Oct 23;9(44):44846–59. doi: 10.1021/acsomega.4c08797 (PMC11541516; doi:10.1021/acsomega.4c08797)
Supplement: Supplementary file 1 — ao4c08797_si_001.pdf [file ao4c08797_si_001.pdf]

SUPPORTING INFORMATION

Gold Nanoparticles for Photothermal and Photodynamic Therapy

Matthew Broadbent, Samantha J. Chadwick, Mathias Brust and Martin Volk*

Department of Chemistry, University of Liverpool, Crown Street, Liverpool L69 7ZD, United Kingdom
* E-mail: m.volk@liverpool.ac.uk

Table of Contents

S1. Experimental Details 2
S1.1. Gold Nanoparticle Synthesis 2
S1.2. UV-vis Absorbance Spectra of Gold Nanoparticles 2
S1.3. Differential Centrifugal Sedimentation 3
S1.4. Cell Culture 3
S1.5. Incubation of Cells with Gold Nanoparticles 4
S1.6. Gold Nanoparticle Uptake by Cells 4
S1.7. Irradiation Experiments 5
S2. Gold Nanoparticle Uptake 6
S2.1. Uptake Quantification Using ICP-OES 6
S2.2. Semi-Quantitative Estimate of Gold Nanoparticle Uptake Using Electron Microscopy 6
S2.3. Semi-Quantitative Estimate of Gold Nanoparticle Uptake Using UV-vis Spectroscopy .. 10
S3. Investigation of HeLa Cell Size at Different Confluencies 13
S4. Cell Control Experiments 15
S4.1. HeLa Cell Viability upon AuNP Incubation 15
S4.2. HeLa Cell Viability upon Laser Irradiation in the Absence of AuNPs 15
S5. Effect of Elevated Temperatures on HeLa Cells 16
S6. Finite Element Simulations of Temperature Profile upon Laser Irradiation 18
S7. Time Course of HeLa Cell Death after Irradiation in Presence of AuNPs 20
S7.1. PTT Conditions 20
S7.2. PDT Conditions 20
S8. Investigation of HeLa Cell Killing upon Irradiation in Presence of AuNPs Using the MTT Assay 21
S8.1. Experimental Details 21
S8.2. Control Experiments 21
S8.3. Results 22
References 23

S1. Experimental Details

S1.1. Gold Nanoparticle Synthesis

Citrate Stabilized AuNPs: 12-15 nm citrate stabilized AuNPs were synthesised by citrate mediated reduction of $HAuCl_4$ using the Turkevich-Frens method.[1] 150 mL of 0.32 mM HAuCl4 solution were refluxed in a round-bottom flask with vigorous stirring for 5 minutes. 4.5 mL of 39 mM aqueous trisodium citrate was added rapidly. The colour changed from pale yellow to colourless, to deep purple and finally to ruby red. The mixture was refluxed for a further 25 minutes and allowed to cool overnight under stirring. The solution was then filtered through fluted filter paper and stored in a fridge.

CALNN-Stabilized AuNPs: Citrate was replaced as stabilizer by the covalently bound pentapeptide with the sequence CALNN, which has been shown to yield highly stable AuNP solutions, using standard methods.[2] Briefly, 100 µL of an aqueous 1 mg/mL solution of CALNN (Peptide Protein Research Ltd.) was added to 900 µL 2 nM citrate stabilized AuNP solution, mixed well and left overnight at room temperature. The obtained nanoparticles were purified from excess ligand by centrifuging 3 times (12,000 rpm, 20 min, 15°C), discarding the supernatant and resuspending in MQ H_2O after each cycle.

S1.2. UV-vis Absorbance Spectra of Gold Nanoparticles

UV-vis absorbance spectra were recorded on different spectrometers (Thermofisher Scientific Genesys 10S, Agilent Cary 8454, Ocean Optics 2000+) using glass or quartz cuvettes with a path length of 1 cm. Figure S1 shows typical spectra of 14 nm citrate and CALNN-stabilized AuNPs, here normalized to the maximum Surface Plasmon Resonance (SPR) absorbance near 520 nm. As expected, the slightly thicker ligand shell of CALNN-stabilized AuNPs results in a minor shift of the SPR band to higher wavelengths.

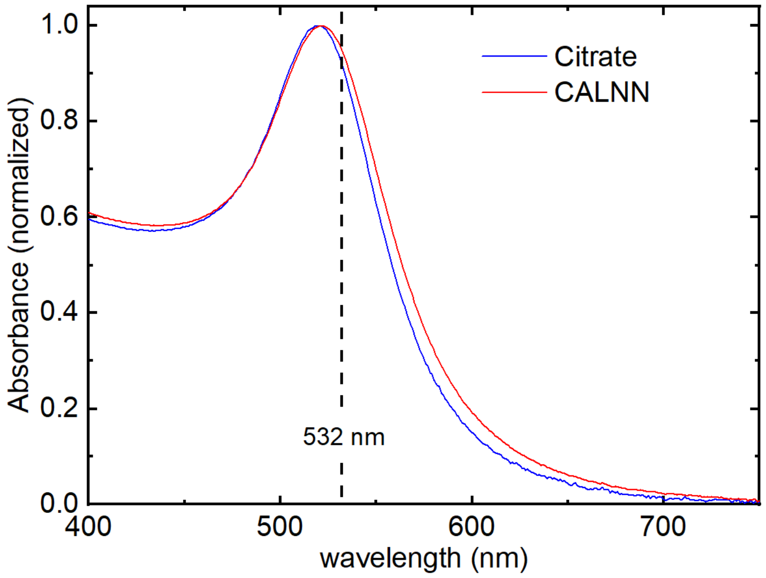

Figure S1. UV-vis absorbance spectra of 14 nm citrate and CALNN-stabilized AuNPs, normalized to the maximum SPR absorbance near 520 nm. The dashed line indicates the wavelength of the laser used for cell irradiation experiments.

S1.3. Differential Centrifugal Sedimentation

AuNP diameters and size distributions were measured using Differential Centrifugal Sedimentation (DCS) in a DC24000 disc centrifuge (CPS Instruments Inc.). Freshly prepared gradient fluids (8-24 wt.% sucrose in MQ H_2O in 9 steps) were filled successively into the disc rotating at 24,000 rpm. 0.377 µm poly(vinyl chloride) particles (Analytik Ltd.) were used before each sample measurement for calibration. Each sample was analyzed three times to verify data reproducibility.

Figure S2 shows typical size distributions for citrate and CALNN-stabilized AuNPs; these are the raw data, from which the absolute AuNP core diameters can be determined with high precision as described previously; in particular, it should be noted that the low density capping layers shift the distribution by 1-2 nm to lower diameters, with the thicker CALNN layer resulting in a larger shift.[3,4] These distributions show that the AuNP samples are highly monodisperse and confirm the successful binding of CALNN.

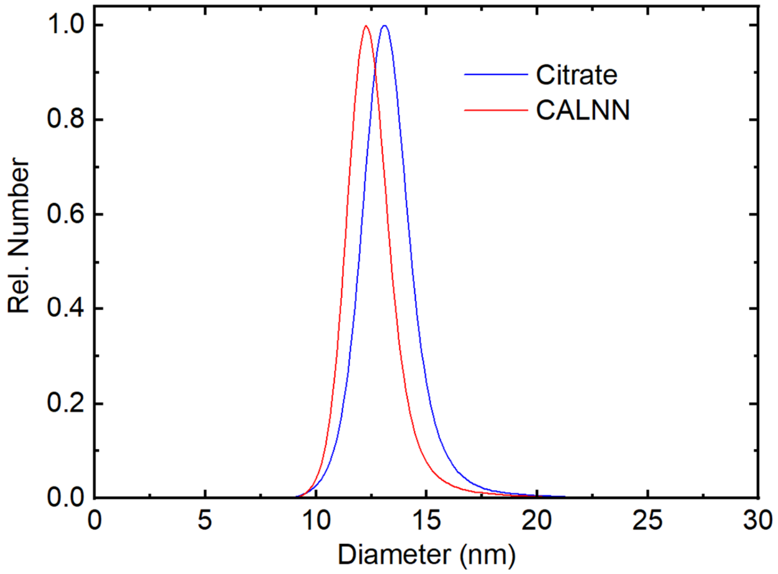

Figure S2. DCS size distributions of citrate and CALNN-stabilized AuNPs prepared from the same batch of AuNPs. These are the raw data, which show that the gold core had a diameter of 13.9 nm.[3,4]

S1.4. Cell Culture

HeLa cells were cultured in cell culture medium (CCM), consisting of Dulbecco's Modified Eagle Medium (DMEM) supplemented with 10% FBS, 1% non-essential amino acids and 1% penicillin streptomycin. Cells were incubated at 37°C in a 5% CO_2 humidified atmosphere. Passages 4-30 were used for experiments and cells were typically grown to 70-80% confluence before splitting. All irradiation experiments presented here were undertaken on cell cultures with at most 70-80% confluence.

S1.5. Incubation of Cells with Gold Nanoparticles

On exposure of citrate stabilized AuNPs to the CCM, the citrate capping layer is displaced by a corona formed from the serum proteins which stabilizes the AuNPs.[5-8] This corona increases gradually during the 24 hour exposure of AuNPs to CCM prior to their application to cell cultures to yield an overall thickness of ~15 nm.[6,7] It is reasonable to suggest that this corona mimics the protein corona which systemically administered AuNPs would pick up in the blood stream. The serum protein corona plays an important role in the uptake of nanoparticles by cells. In particular, it prevents their aggregation in cell medium.[5-7] However, it also appears to be important for the uptake itself, since in the absence of serum citrate-stabilized AuNPs do not interact with cells;[9] similarly, AuNPs with a dense PEG capping layer, which prevents serum protein corona formation,[4,10] are essentially not taken up by cells even in the presence of serum.[10,11] On the other hand, serum proteins are in competition with AuNPs for binding to receptors, so that an increase of the serum concentration leads to a decrease of binding to the cell surface [12], and thus of receptor-mediated endocytosis, which has been shown to be the dominant mechanism for the uptake of AuNPs by HeLa cells.[5,11,13-15] In general, the amount and composition of the protein corona can greatly affect the mechanism and amount of nanoparticle uptake.[16] We therefore took care to use identical incubation protocols for AuNP uptake and irradiation experiments described here, so that their results can be directly related to each other.

To ensure reproducible formation of the protein corona, AuNP solutions were mixed 1:1 with CCM to achieve a final AuNP concentration of 2 nM or 4 nM and left standing for 24 hours prior to their use for HeLa cell incubation. This mixture was then added to freshly rinsed (PBS) HeLa cells in 35 mm or 100 mm cell culture dishes (prepared on the previous day to allow for complete cell attachment) and left in the incubator (37°C, 5% CO_2) for 3 or 24 hours. The cells were then washed 3 times with phosphate buffered saline (PBS), pH 7.4.

S1.6. Gold Nanoparticle Uptake by Cells

For quantitative AuNP uptake analysis using Inductively Coupled Plasma Optical Emission Spectrometry (ICP-OES), the cells were incubated with AuNPs as described above, then repeatedly washed with PBS (pH 7.4), detached using 1 mL trypsin and diluted to 10 mL with DMEM. The total number of cells in the suspension was determined by counting the cells in a small aliquot with a hemocytometer, the suspension was then centrifuged and the supernatant removed to give a cell pellet which was dissolved in 0.5 mL aqua regia over 3 days. The solution was diluted to 5 mL with MQ-water and submitted for ICP-OES analysis.

For obtaining TEM images, cells in a 35 mm culture dish were washed with warmed PBS before being fixed with a 1 mL solution containing 4% paraformaldehyde and 2.5% glutaldehyde (taken from 16 and 25% stock solutions, respectively) in 0.1 M PBS, pH 7.4 for 1 hour. The cells were then rinsed with room temperature 1 mL PBS twice and post-fixed using reduced OsO_4 (2% OsO_4, 1.5% potassium ferrocyanide in PBS) by adding a few drops to cover the bottom of the dish for 1 hour before being washed 5 times with 1 mL MQ-H_2O for at least 3 minutes per wash. 1 mL aqueous 2% OsO_4 solution was added to the cells for 40 minutes and a second set of 5 x 1 mL MQ-H_2O washes was performed before leaving the cells in 1 mL 1% aqueous uranyl acetate solution at 4°C overnight. On the second day the cells were washed with MQ-H_2O (3 x 1 mL, 15 minutes washes) and dehydrated gradually using a series of ethanol solutions (1 mL) in water (30, 60, 70, 80 and 100%) and finally the cells were embedded in epoxy resin. The resin was polymerised at 60°C for at least 48 hours. Ultrathin sections (*ca.* 70 nm) were cut using a diamond knife on a Leica Ultramicrotome and mounted on piloform coated 200 hexagonal mesh copper grids. The sections were then post stained with 5% uranyl acetate in 50% ethanol and 2% aqueous lead citrate solution and imaged with a FEI Tecnai Spirit TEM at 120kV or a FEI 250 Quanta FEG-ESEM at 30kV in STEM mode.

S1.7. Irradiation Experiments

HeLa cells were incubated with AuNPs as described above. At the end of the incubation time, prior to the removal of the CCM:AuNP solution, several areas on the dish were imaged using a microscope (GX Optical Microscope). After repeated washing and immersion in PBS (pH 7.4), the cells in some of those areas were irradiated at room temperature with a cw 532 nm diode pumped solid state Opus Quantum Laser with a $1/e^2$ beam diameter of 1.85 mm and a power of up to 3 W. This maximum power corresponds to a maximum intensity of 223 W/cm^2 and an average intensity of 210 W/cm^2 in the central circular area with a diameter of 0.5 mm which was used for quantitative analysis since the laser intensity drops by less than 14% from the maximum in this area. A micrometer stage and a marker on the cell dish were used for reproducible imaging and irradiation of the correct areas. After irradiation, the cells were placed under CCM and incubated at standard conditions (37°C, 5% CO_2) for 24 hours (unless otherwise stated), then cell viability was assessed using trypan blue. For this, the CCM was replaced by a 1:1 (v/v) mixture of CCM:trypan blue solution (0.4%) and the dish was returned to the incubator for 5 minutes. The cells were then washed twice with PBS, 1 mL of CCM was added to the dish and the cells were imaged using the microscope. The number of live cells, identified by the absence of trypan blue staining or significant morphological changes, was counted manually in a circular area with a diameter of 0.5 mm around the center of the laser beam for the images taken prior to and 24 hours after irradiation using the “Point” tool in ImageJ;[17] an example of cell counting is shown in Figure S3. These numbers were used to calculate cell viability, by comparing their ratio to the expected growth rate, obtained from a non-irradiated control area on the same dish:

$$viability = \frac{live\ cells\ 24\ hours\ after\ irradiation}{live\ cells\ before\ irradiation\ \times\ growth\ rate}$$

$$growth\ rate = \frac{live\ cells\ in\ control\ area\ after\ 24\ hours}{live\ cells\ in\ control\ area\ before\ irradiation}$$

All irradiation experiments were repeated several times and the results are reported as the average viability ± standard deviation.

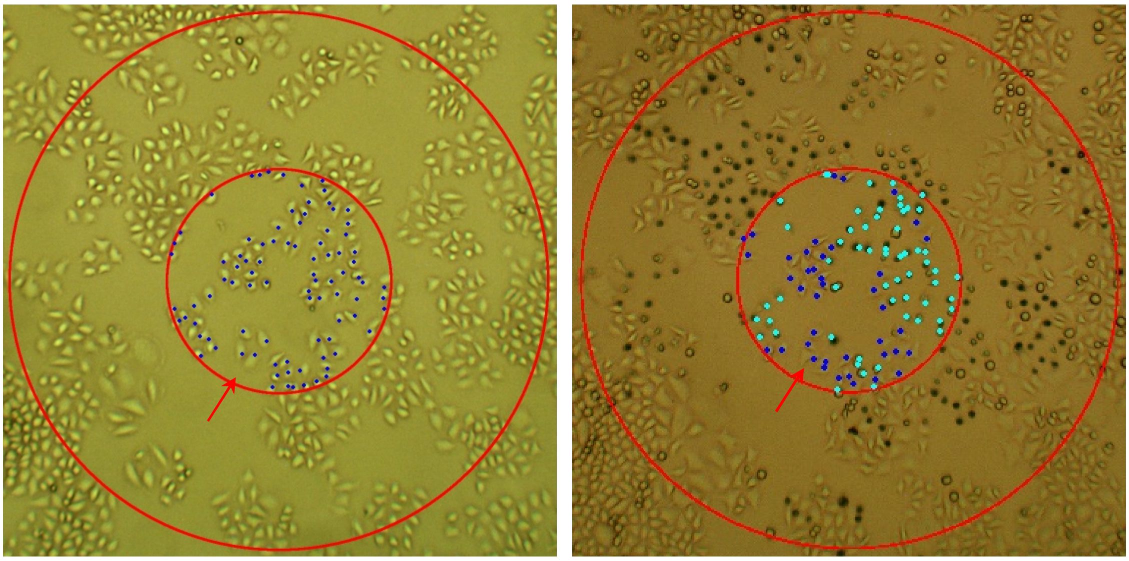

Figure S3. Microscope images of HeLa cells, incubated with 2 nM citrate stabilized AuNPs for 3 hours, before (left) and 24 hours after (right) irradiation with 0.6 W for 15 minutes. The red circles have diameters of 0.5 mm and 1.2 mm, respectively. The dark blue dots mark live cells in the inner circle, identified by the absence of staining or significant morphological changes, whereas the light blue dots mark dead cells. Significant cell splitting has occurred outside and around the outer perimeter of the irradiated area, whereas in general not much cell splitting was found in the inner circle, with one exception, indicated by the red arrow).

S2. Gold Nanoparticle Uptake

S2.1. Uptake Quantification Using ICP-OES

Table S1. AuNPs associated with cells (internalized in endosomes or bound to the cell surface). For this analysis, only dishes with >1000 μm²/cell available area were considered. AuNP@CALNN were only tested under lower confluency conditions and only in smaller dishes, resulting in a larger variation of the results because of the smaller number of cells incubated.

	Incubation Conc. / nM	Incubation time / h	AuNPs/cell	+/- stdev	Number of experiments
AuNP@citrate	2	3	45,000	5,600	15
	2	24	352,000	49,000	4
AuNP@CALNN	2	3	75,000	9,000	7
	4	3	220,000	48,000	5
	4	24	590,000	21,000	2

S2.2. Semi-Quantitative Estimate of Gold Nanoparticle Uptake Using Electron Microscopy

An attempt was made to quantify the number of AuNPs per cell using TEM/STEM. For this purpose, a cell culture section (thickness of *ca.* 70 nm) was imaged by TEM/STEM and a particular cell selected; then, individual images of this cell were taken at high enough magnification so that individual AuNPs could be seen, making sure that the full cell area was imaged at this resolution. The AuNPs in that section of the cell were counted and their number multiplied by 5000/70 to estimate the total number of AuNPs for this cell, accounting for a HeLa cell height of 5 μm.

Figs. S4 and S5 show all individual images which were found to contain AuNPs for two particular HeLa cells, respectively, after incubation with 14 nm citrate-stabilized AuNPs for 3 hours, indicating the approximate number of AuNPs in each endosome or bound to the surface. From these, it can be estimated that the total number of AuNPs associated with these cells (taken up by endocytosis or bound to the surface) are approx. 25,500 and 48,500, respectively, with approx. 90% of those AuNPs found in the cell interior. This is in semi-quantitative agreement with the ICP-OES result of 42,000 NPs per cell, taking into account the uneven distribution of NPs through the cell sections, the difficulty in counting NPs in densely packed endosomes and the averaging of the value in one section over the whole cell.

Fig. S6 shows all individual images which were found to contain AuNPs for a HeLa cells after incubation with 14 nm CALNN-stabilized AuNPs for 3 hours. There are more NPs attached to the cell membrane than after incubation with citrate stabilized AuNPs, with approximately 230 AuNPs in endosomes in this section and ~1800 NPs attached to the cell membrane, resulting in an estimate of a total number of 145,000 AuNPs associated with this cell, which again is in semi-quantitative agreement with the ICP-OES results.

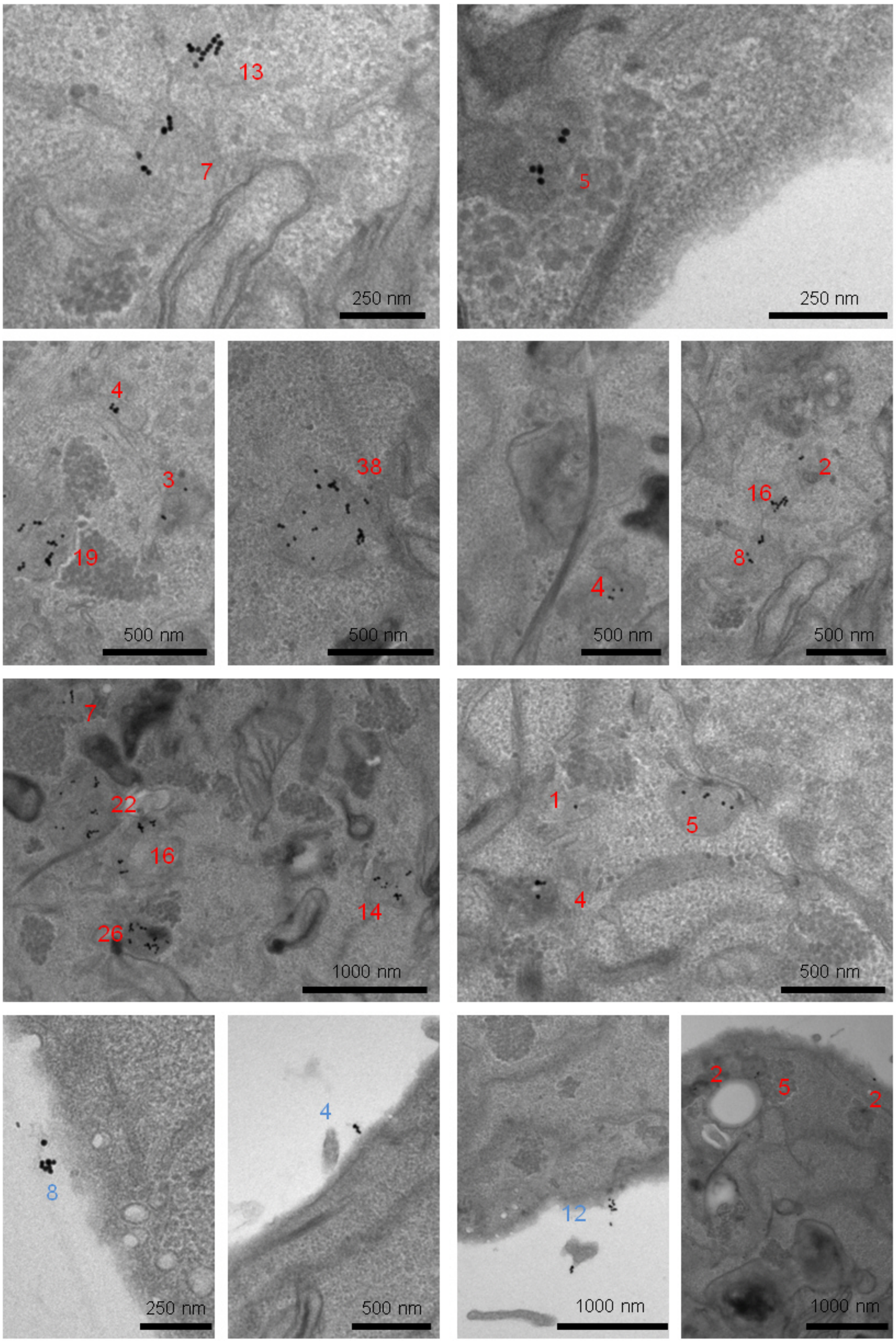

Figure S4. TEM images of a HeLa cell after incubation with 14 nm citrate stabilized AuNPs (3 hours, 2 nM). The images show all parts of the cell sections containing AuNPs. The numbers are the amount of AuNPs in endosomes (red) or attached to the cell surface (blue). Figure continued on next page…

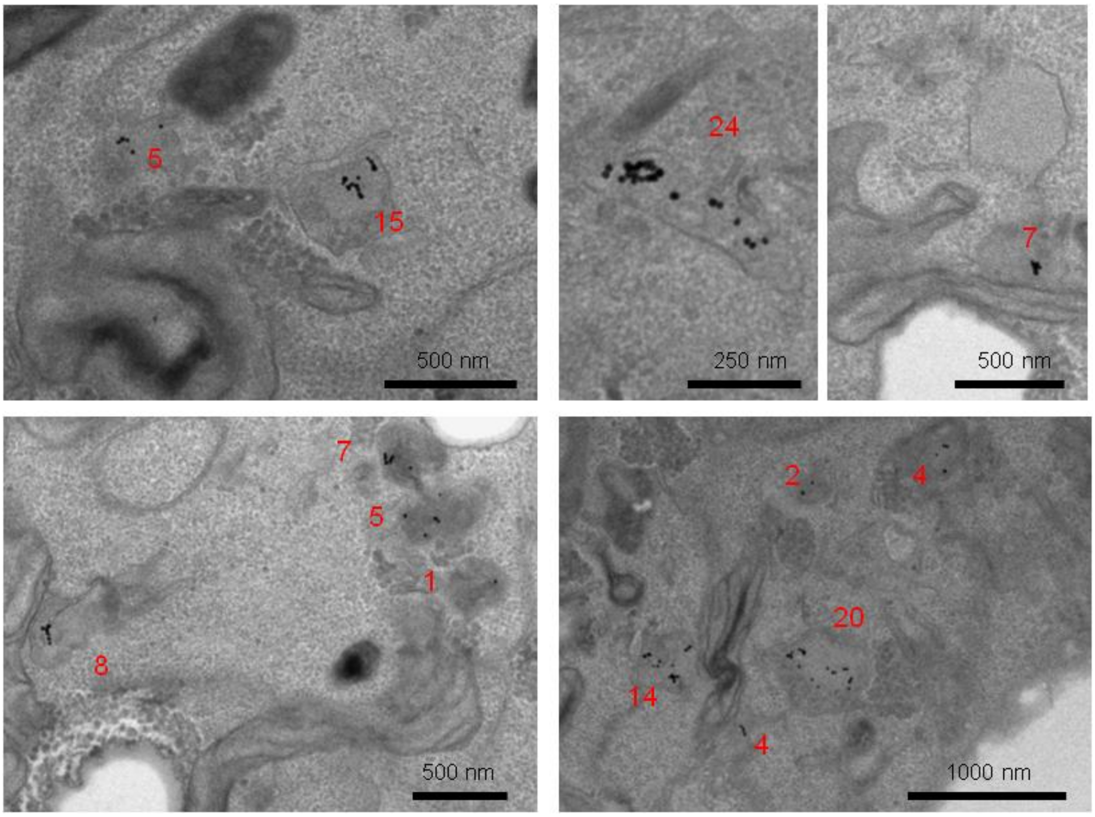

Figure S4 (continued). TEM images of a HeLa cell after incubation with 14 nm citrate stabilized AuNPs (3 hours, 2 nM). The images show all parts of the cell section containing AuNPs. The numbers are the amount of AuNPs in endosomes (red) or attached to the cell surface (blue). Overall, this cell section contained a total of 363 AuNPs, with 339 found in the cell interior and 24 bound to the surface.

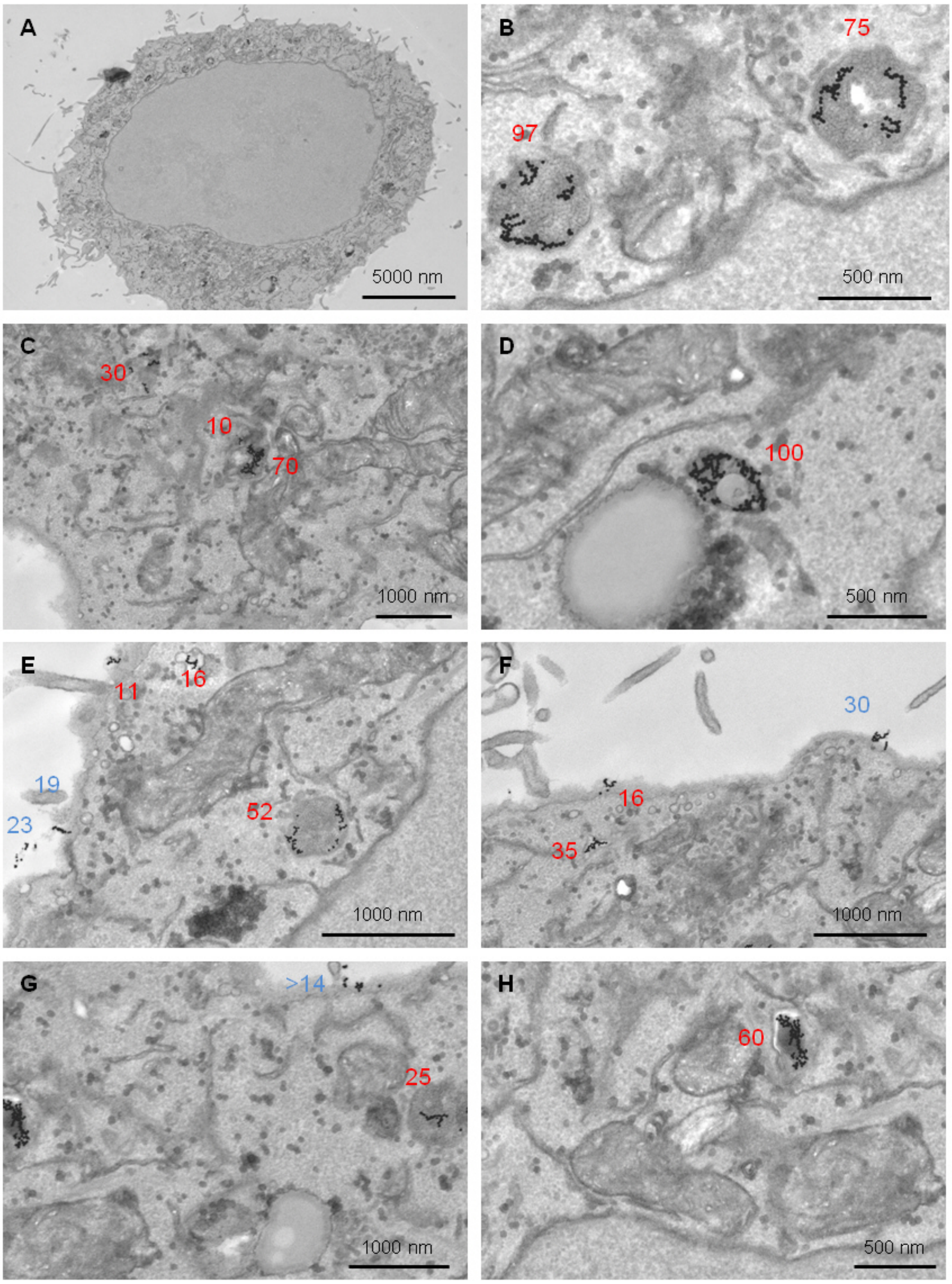

Figure S5. STEM images of a HeLa cell after incubation with 14 nm citrate stabilized AuNPs (3 hours, 2 nM). A) The whole cell section at low magnification; B-H) all parts of the cell section containing AuNPs at higher magnification. The numbers are the amount of AuNPs in endosomes (red) or attached to the cell surface (blue). Overall, this cell section contained a total of 683 AuNPs, with 597 found in the cell interior and 86 bound to the surface.

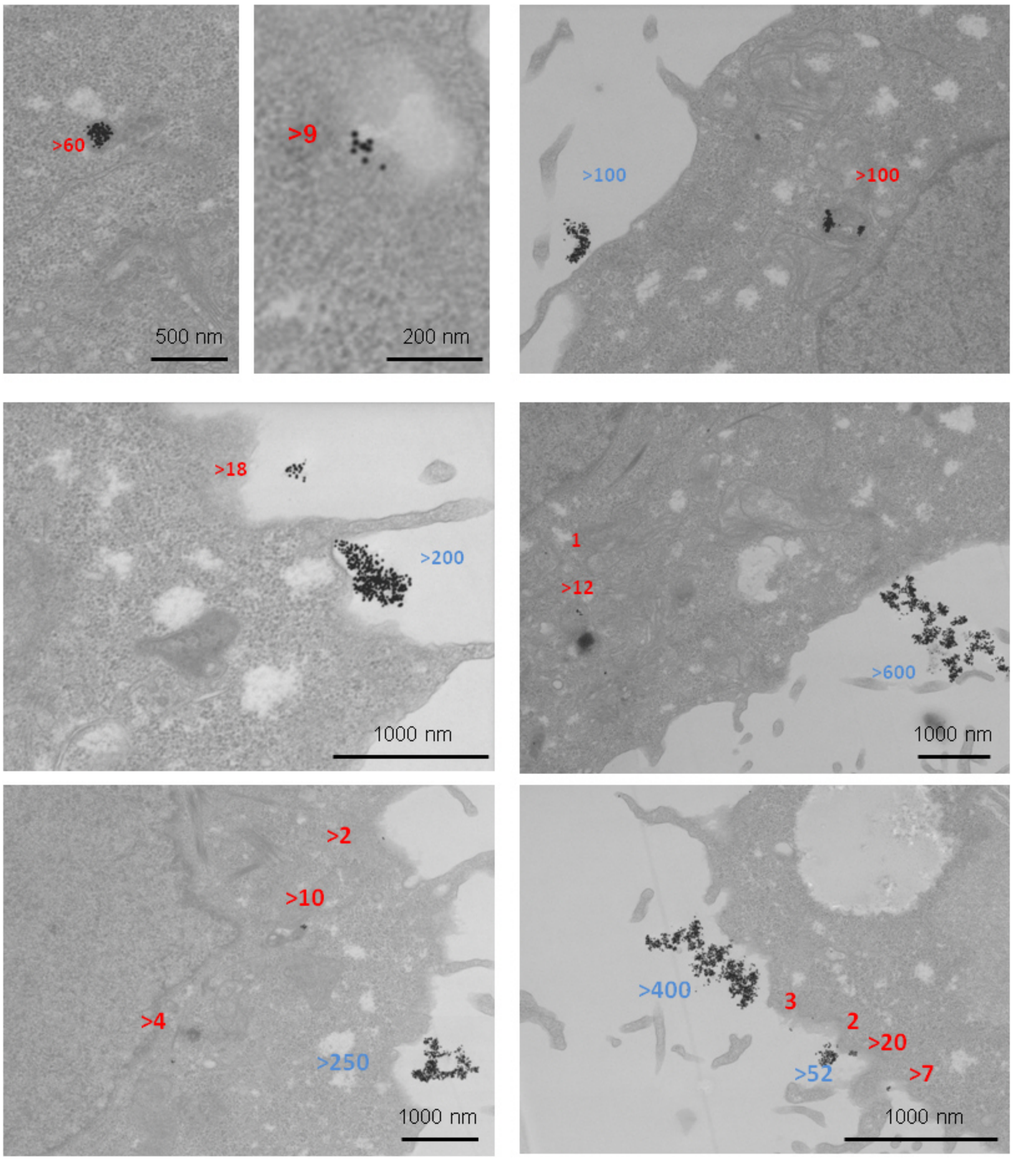

Figure S6. STEM images of a HeLa cell after incubation with 14 nm CALNN-stabilized AuNPs (3 hours, 4 nM). The images show all parts of the cell section containing AuNPs. The numbers are the amount of AuNPs in endosomes (red) or attached to the cell surface (blue). Overall, this cell section contained a total of approximately 2030 AuNPs, with 230 found in the cell interior and ~1800 bound to the surface.

S2.3. Semi-Quantitative Estimate of Gold Nanoparticle Uptake Using UV-vis Spectroscopy

UV-vis absorbance spectra of cell cultures containing AuNPs were measured using a home-built setup consisting of a halogen lamp illuminating the cell dish from underneath and an Ocean Optics USB2000 spectrometer with an optical fibre terminating above the cell culture dish. After incubation of HeLa cells in a 35 mm cell culture dish with AuNPs, the cells were washed three times with 1 mL PBS before 600 μL of fresh PBS was added to the dish. The absorbance was calculated using 600 μL of PBS in a 35 mm culture dish as a reference sample.

HeLa cells in a 35 mm culture dish at almost full confluency (~1×10^5 cells/cm^2) were incubated with CALNN-stabilized AuNPs (4 nM, 3 hours). Figure S7A shows the UV-Vis absorbance spectra taken on two such samples, together with microscopic images of the cell cultures. The spectra clearly show the AuNP SPR band, providing further evidence of the presence of AuNPs. The band is broader than that of AuNPs in solution (Figure S1), and the maximum is shifted from 520 to approx. 535 nm. This is expected as the electron microscopy images show that the AuNPs are aggregated in densely packed endosomes or on the cell surface. Precise quantification of the

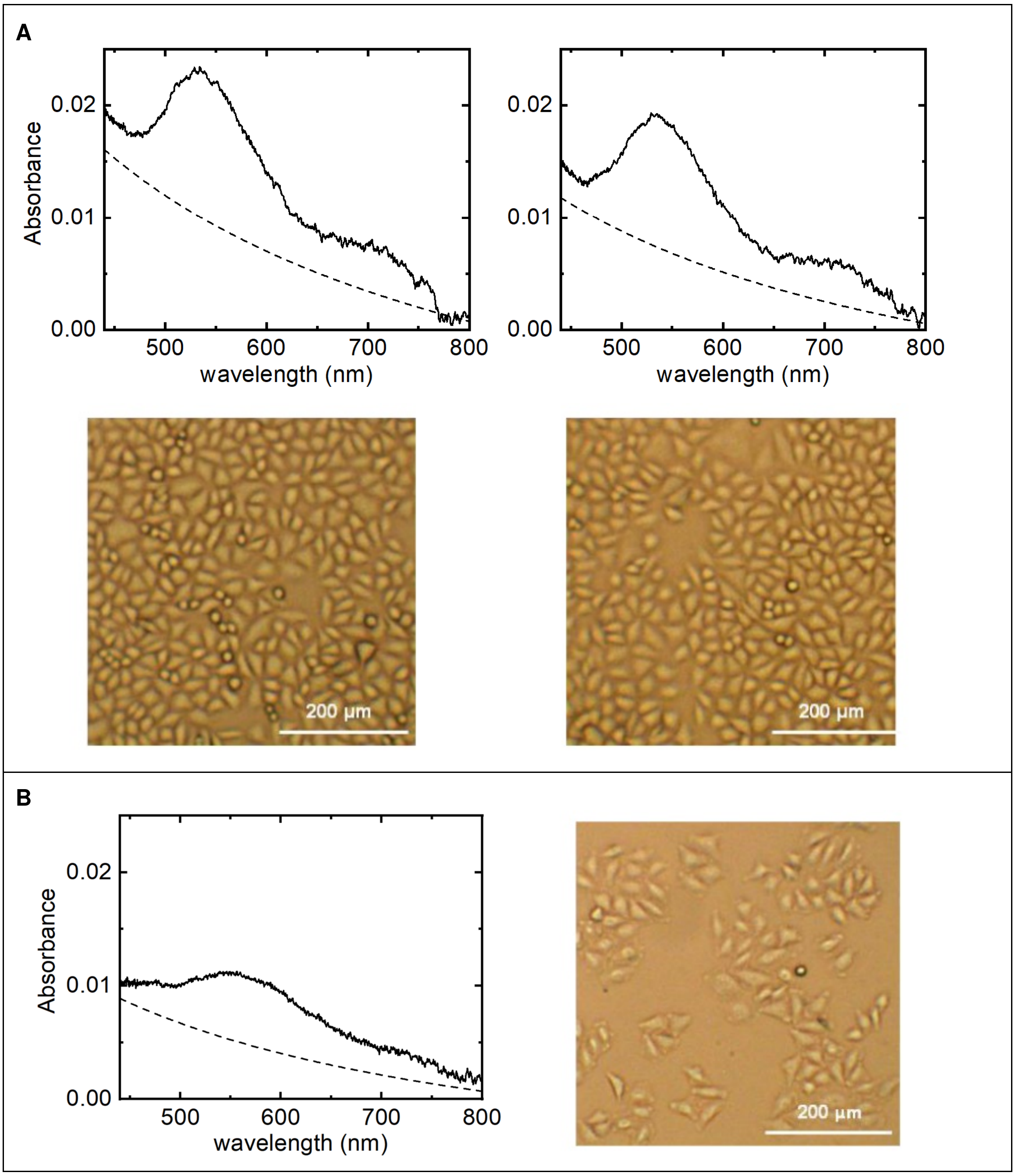

Figure S7. UV-Vis spectra and microscopic images of HeLa cell cultures grown to almost full confluency of ~1×10^5 cells/cm^2 (A), or to less than half of full confluency (B), incubated with CALNN-stabilized AuNPs (4 nM, 3 hours). The dashed lines indicate the scattering background.

absorbance is hindered by the wavelength dependent scattering component arising from the optically inhomogeneous cell culture, which is approximatively shown by the dashed lines, yielding an approximate value of the SPR absorbance in these samples of 0.01. For a sample with lower cell density (~0.5×10^5 cells/cm^2), a correspondingly lower value of ~0.005 was found (Figure S7B).

The expected absorbance of these HeLa cell monolayers can be estimated using the number of cells per cm^2, the number of AuNPs per cell (220,000, as determined by ICP-OES for HeLa cells incubated with 4 nM CALNN stabilized AuNPs for 3 hours (Table S1) and the extinction coefficient of 14.8 nm AuNPs (ε = 3.4x10^8 M^{-1}cm^{-1}), yielding values of 0.011 and 0.005 for the cells at higher or lower confluency, respectively, which agrees reasonably well with the measured values.

In contrast, no SPR absorbance peak was detected for a HeLa cell culture at essentially full confluency after incubation with citrate stabilized AuNPs (2nM) for 3 hours, Figure S8. This again is in good agreement with the predicted absorbance of less than 0.002, which is so small due to the significantly lower uptake of those AuNPs.

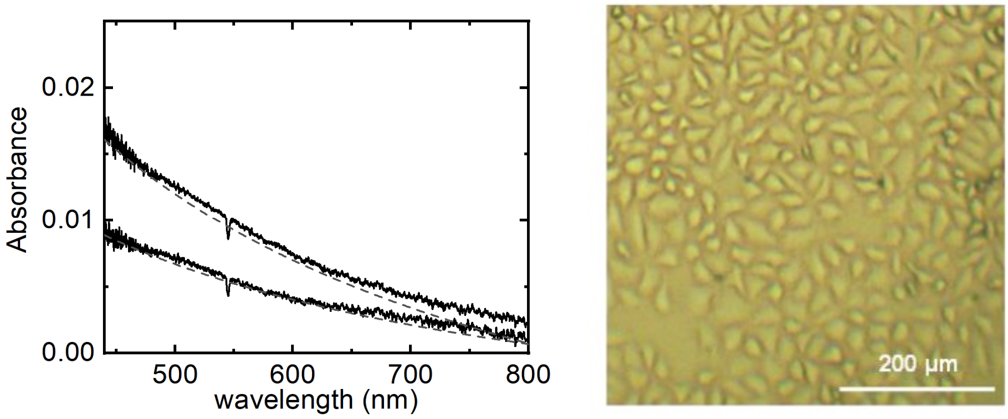

Figure S8. UV-Vis spectra (recorded at different locations) and microscopic image of a HeLa cell culture grown to full confluency ~1×10^5 cells/cm^2, incubated with citrate stabilized AuNPs (2 nM, 3 hours). The dashed lines indicate the scattering background, which is highly variable across the cell dish.

S3. Investigation of HeLa Cell Size at Different Confluencies

In order to determine the average surface area of irradiated HeLa cells, the cells were imaged before irradiation; this was done with the cells in the CCM:AuNP solution rather than PBS as cells change their morphology upon exposure to PBS ("balling up"). Cells within a circle with a diameter of 0.5 mm in the centre of the area to be irradiated, corresponding to the are to be used for quantitative analysis, were traced manually and the occupied area measured using ImageJ,[17] see Figure S9. This area was divided by the number of cells in the circle to give the average area occupied per cell.

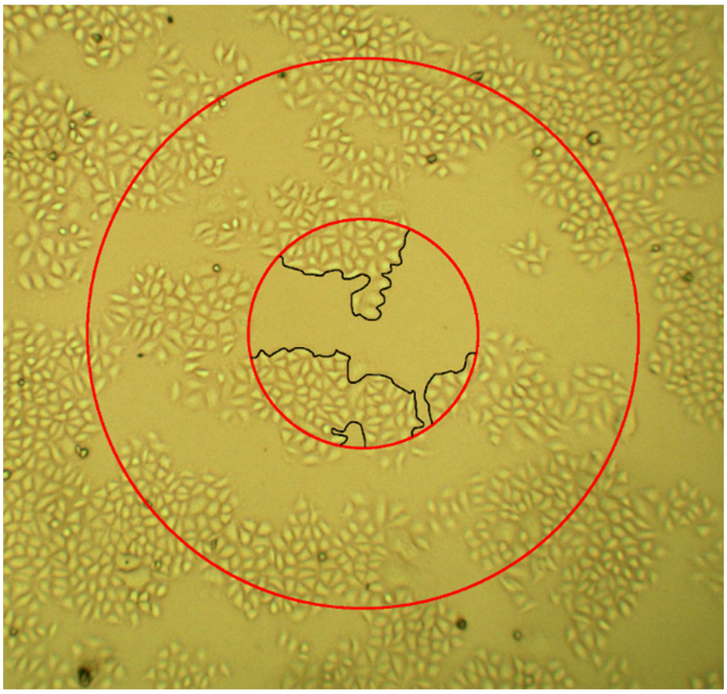

Figure S9. Microscopic image of a HeLa cell culture before irradiation. The red circles have diameters of 0.5 mm and 1.2 mm, respectively, and the black lines show the manual tracing of the outline of the areas occupied by cells used for determining the average area per cell.

Figure S10 shows the results of this analysis for the microscopic images of all irradiated areas just before irradiation. For each analyzed area (i.e. the circle with a diameter of 0.5 mm around the center of the laser beam), the cells were counted and the area occupied by these cells measured. For conditions with a low number of cells in the irradiated area, the average cell size was found to be on the order of 1500 μm^2, similar to the cell area reported for other epithelial cell lines.[18] Under these conditions, an increase in the number of cells in the irradiated area results in only a slight decrease of the average area occupied by each cell, which shows that under these conditions additional cells can make use of the free space available and essentially grow to their unrestricted size. On the other hand, for cell cultures with high confluency, i.e. a larger number of cells in the analyzed area, the average area occupied by a cell decreases significantly since cells are competing for space. The transition occurs in the region where the total area occupied by cells approaches approximately half of the available area (at ~100 cells in the 0.5 mm circle). At a total number of ~200 cells in the circle, the cell culture becomes fully confluent, so that the area per cell is limited by the total area available. For the highest confluencies used here, the average area per cell is found to be only around 600 μm^2.

However, the spread of the data points, and the selected images, also show that local fluctuations of the cell density limits the correlation between the average cell size and the number of cells in the analyzed area. For example, the image with 110 cells (cyan) shows a much better spread of those cells over the available area than is the case for the image with 121 cells (blue), where there is a large empty cell dish area in spite of the larger number of cells, so that these images yield very different area/cell results.

Since our AuNP uptake results indicate that AuNP uptake is correlated with the accessible cell surface, in line with previous literature reports,[19-22] it is necessary to explicitly measure the average area occupied by cells for each area analyzed to correct for this local confluency variation. Only doing this allowed us to verify that the large variation of cell viability after incubation with citrate stabilized AuNPs for 3 hours and irradiation at 210 W/cm^2 for 3 minutes is to a large extent arising from this local fluctuation of cell density and the ensuing variation of cell size (Figure 6). Attempts to correlate viability with the number of cells in the analyzed area, which is a simpler measure of cell density, were not successful.

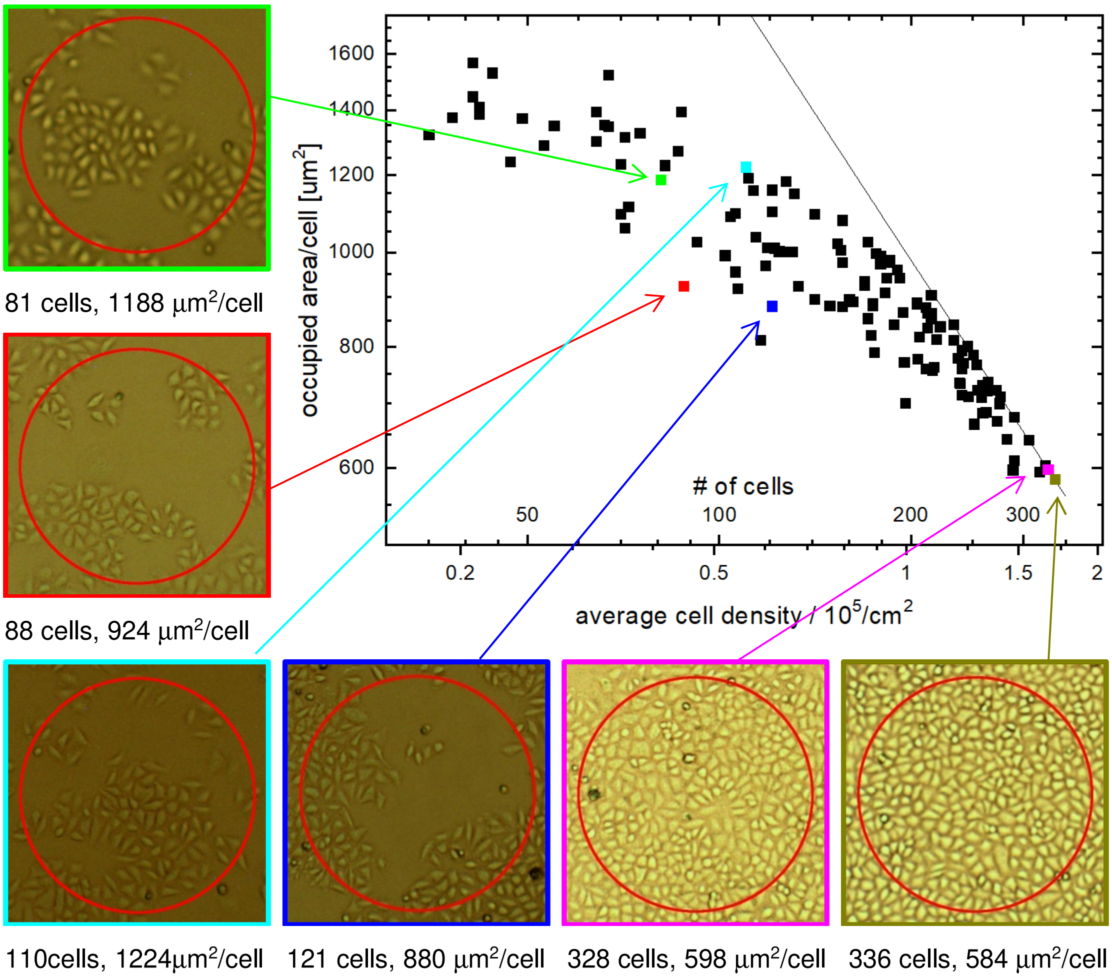

Figure S10. Average surface area of cells in the center of the irradiated cell culture areas vs. the number of cells in the center circle with 0.5 mm diameter used for viability analysis. The solid line is the total area available for each cell, i.e. the area of the circle divided by the number of cells. The images show selected examples at different confluencies, the red circles (0.5 mm diameter) indicate the analyzed areas.

S4. Cell Control Experiments

S4.1. HeLa Cell Viability upon AuNP Incubation

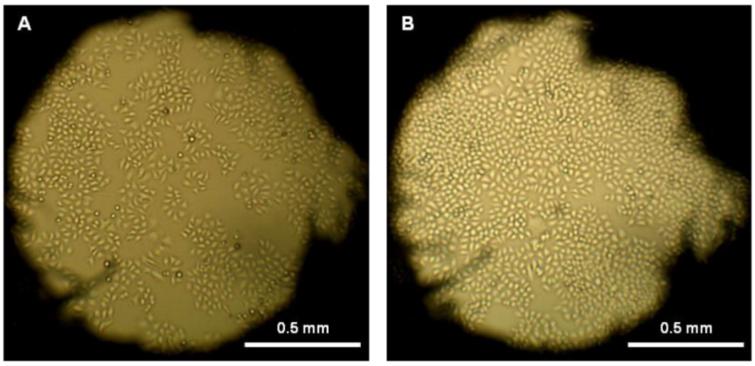

Figure S11. HeLa cells in a non-irradiated control area; (A) immediately after incubation with 2 nM citrate-stabilized 14 nm AuNPs for three hours; B) 24 hours later, after trypan blue treatment. No staining of cells is visible, indicating that all cells had an intact cell membrane at this time. A quantitative analysis shows that the cell growth rate, i.e. the increase of the number of cells over 24 hours, is slightly reduced after incubation with citrate-stabilized AuNPs for 3 hours, compared to cell dishes which were exposed to a 1:1 (v/v) mixture of CCM and milli-Q water for the same time, from (2.05 $\pm$ 0.06) to (1.68 $\pm$ 0.19), in agreement with previous reports for the incubation of HeLa cells with citrate-stabilized AuNPs under similar conditions.[7,23] In light of the absence of trypan blue staining, it is tempting to ascribe this effect to a slightly reduced rate of cell splitting with no direct cytotoxicity. However, it is possible that the effect results from a few cells undergoing apoptosis, which are not stained by trypan blue, rather than a reduced rate of cell splitting. This suggestion is based on the observation that the effect of citrate AuNPs on the proliferation of human dermal fibroblasts, which is similar to the one observed here for HeLa cells, is due to cells undergoing apoptosis.[24] The same study also showed that once 13 nm AuNPs are removed from the CCM, non-apoptotic cells rapidly recover even after prolonged exposure to AuNPs.

S4.2. HeLa Cell Viability upon Laser Irradiation in the Absence of AuNPs

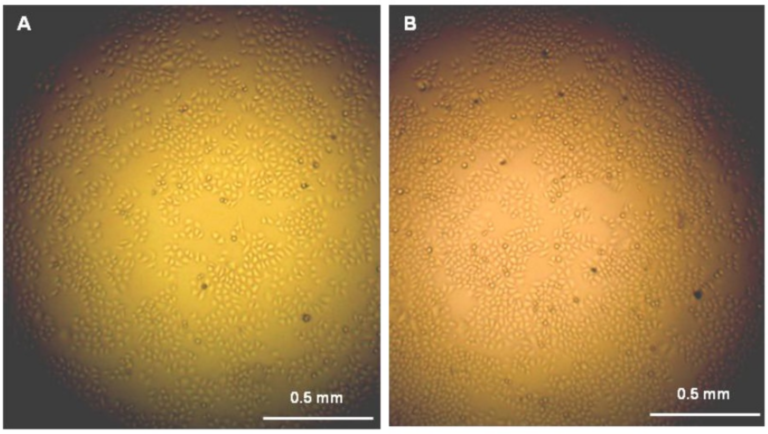

Figure S12. HeLa cells in the absence of AuNPs; (A) immediately after incubation with a 1:1 mixture of water and CCM for three hours, prior to irradiation and (B) 24 hours after irradiation with 532 nm light at an intensity of 210 W/cm^2 for 5 minutes, immediately after trypan blue treatment.

Table S2. HeLa cell viability after irradiation in absence of AuNPs under different conditions.

Intensity / Wcm^{-2}	Irradiation time / min	Cumulative dose / $W cm^{-2}$ min	viability
210	5	1050	0.74 ± 0.06
210	3	630	0.85 ± 0.08
140	3	420	0.91 ± 0.02
70	3	210	0.90 ± 0.06
210	3	630	0.85 ± 0.08
42	15	630	0.67 ± 0.04
21	30	630	0.55 ± 0.07

S5. Effect of Elevated Temperatures on HeLa Cells

HeLa cells cultured in a 35 mm dish were taken from the incubator, the cell culture medium removed and 2 mL PBS (pH 7.4) added to mirror the incubation conditions used during irradiation. The dish was sealed with parafilm and partially submerged in a water bath set to different temperatures for 5 minutes. Cell viability was determined after 24 hours using the trypan blue assay.

It is important to note that immersion of the dish in a water bath does not result in immediate temperature equilibration due to limited thermal flow through the dish material, although the use of a water bath is expected to yield faster heating of the cell culture than the use of an oven. For verifying temperature equilibration, a thermocouple was clamped to the bottom of a dish that contained 2 mL PBS and the temperature was recorded continuously, see Figure S13. This shows that it takes less than 3 minutes for the cells to get to within 1°C of the target temperature, which is somewhat slower than the heating achieved using irradiation of AuNPs that are associated with the cells, compare Figure S15.

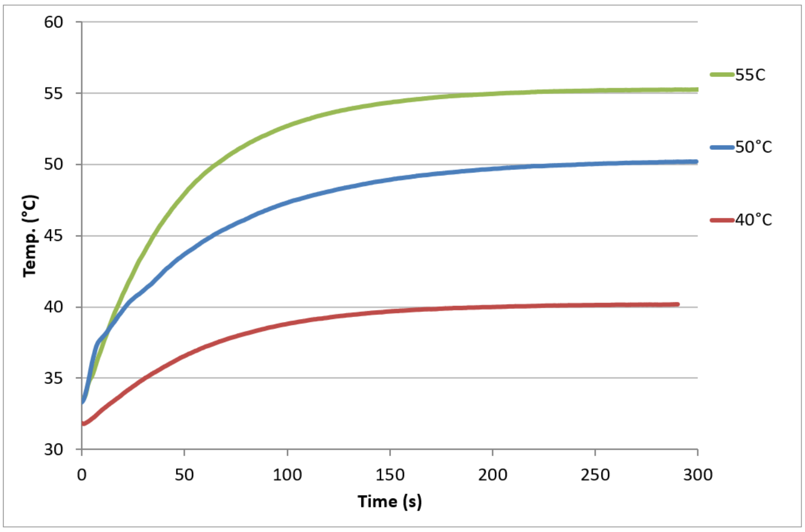

Figure S13. Time dependence of the temperature at the bottom of a 35 mm cell culture dish containing 2 mL PBS upon immersion in a water bath which is at the temperature indicated.

Figure S14 shows typical results of those experiments. When the water bath was set to 40°C, so that the cells were at a temperature of 39-40ºC for at least 3 minutes, they survived and normal cell division occurred over night. However, keeping the cells at a temperature closer to 50 minutes resulted in decreased viability; although most cells seem to be alive after 24 hours, they did not divide during this time and some cells seem to have died and detached, resulting in an overall viability of less than 0.5 when comparing this outcome to the normal growth rate of 2.05 ± 0.06. An incubation temperature of 55ºC, on the other hand, leads to an almost complete loss of cells and a viability of less than 0.1.

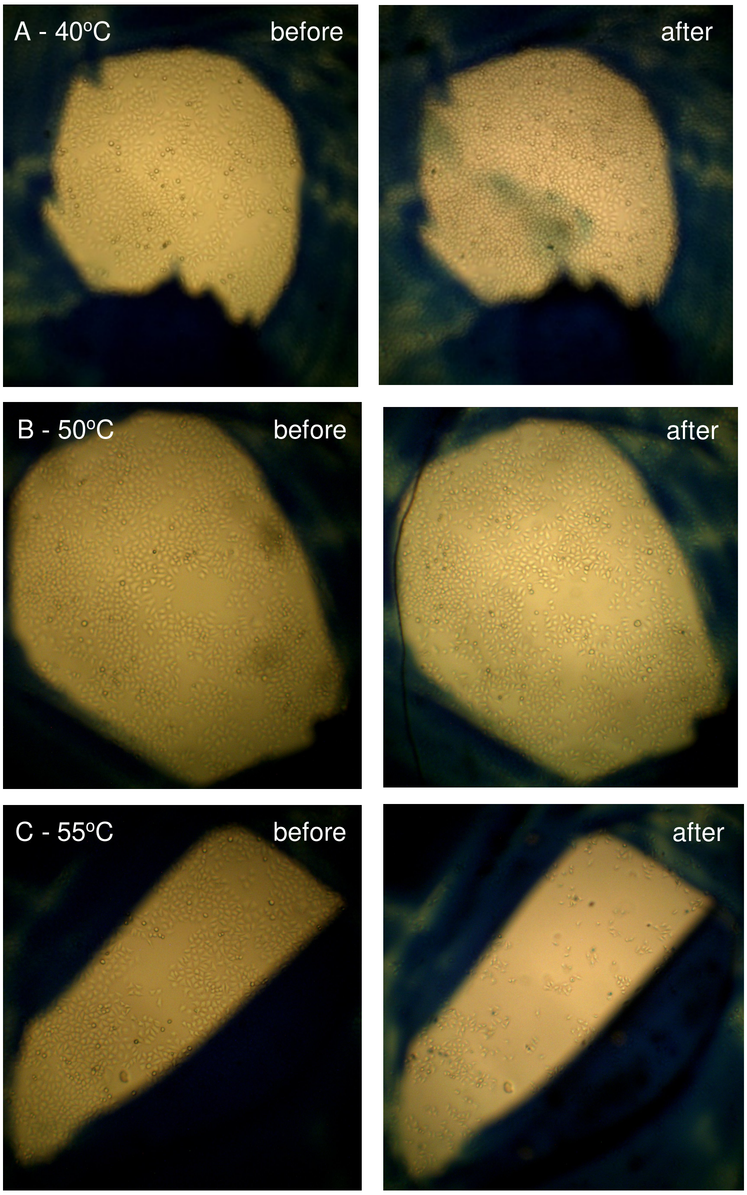

Figure S14. Microscopic images of HeLa cell cultures before (left) and 24 hours after (right) incubation in a water bath at 40ºC (A), 50ºC (B) or 55ºC (C) for 5 minutes.

S6. Finite Element Simulations of Temperature Profile upon Laser Irradiation

The spatio-temporal temperature distribution of the buffer solution above a thin layer of AuNP-loaded cells on a culture dish during irradiation with a laser beam was calculated using finite-element dynamic heat transport simulations based on the Fourier equation;[25-27]] experimental observations on phantom models containing AuNPs have verified this approach.[26,27] These simulations used the same code as the simulations described in a previous publication,[15] although the parameters were adjusted to the current experimental conditions. Only a brief summary of those simulations is given here.

The extinction coefficient of spherical AuNPs[28] at 532 nm (e.g. $\varepsilon = 2.7 \times 10^8$ M^{-1} cm^{-1} for 14 nm AuNPs) allows the calculation of the absorption cross section, $\sigma = \varepsilon \ln 10 / N_A$, where N_A is Avogadro's constant, and thus of the rate of absorption of light energy by a single AuNP at a given light intensity I, $\mathrm{d}Q_{NP}/\mathrm{d}t = \sigma \times I$. The extinction coefficient of spherical AuNPs of this size is dominated by absorption, with only a negligible contribution from scattering,[29] so that this calculation based on the extinction coefficient indeed is correct; for the same reason, it is not necessary to trace scattered rays and account for their absorbance. AuNPs release this absorbed energy very rapidly to the surrounding aqueous solution in the form of heat, on the time scale of ~50 ps for AuNPs with 14 nm diameter.[30]

For calculating the total heating power per unit volume, we assume a uniform distribution of the AuNPs in a layer with a thickness of 5 µm, i.e. the typical height of HeLa cells, where the density of AuNPs is calculated from the density of cells in a particular irradiated area (determined by manual counting) and the number of AuNPs taken up by each cell (Figure 1A in the main text).[*] The spatial distribution of the heating power is determined by the Gaussian distribution of the laser intensity (TEM_{00} mode with $1/e^2$ diameter of 1.85 mm). This position-dependent heating power per unit volume, $\mathrm{d}q/\mathrm{d}t$, which is non-zero only in the 5 mm thick cell layer, was used as the source term in the Fourier heat equation, which applies throughout the buffer:

$$\rho c \frac{\partial T}{\partial t} = \kappa \nabla^2 T + \frac{\mathrm{d}q}{\mathrm{d}t} \quad \text{(S1)},$$

where T denotes the time- and position-dependent temperature, t the time, and κ the heat conductivity, ρ the density and c the heat capacity of the medium, which were approximated by the values for neat water, since virtually all heat transfer on the relevant length and time scales takes place in the buffer solution above the cells and not within the cells themselves. The Fourier equation only accounts for conductive heat transfer and ignores convection, which is valid as long as the temperature difference between the cell layer and the top of the water column is less than 15°C, since then the Rayleigh number is below the critical Rayleigh number.[31,32] For conditions where higher temperatures are achieved, convective heat transfer cannot be completely ruled out, which would result in faster heat removal and thus in a somewhat lower temperature of the cell layer than estimated here; however, since this would be expected to become relevant only once the temperature of the cell layer rises well above 40°C, this does not affect the main conclusions described here.

The simulations were implemented using a finite-element method with discrete time and spatial steps, making use of the cylindrical symmetry of the system. The total volume considered in these calculations was a cylinder of 12 mm radius and 2.5 mm height, the latter corresponding to the depth of the buffer solution above the cell culture; heat flow from the solution to air was as-

---

[*] Confluency in the irradiated area was not more than 80% for all experiments, but it cannot be ruled out that the use of AuNP uptake numbers obtained under low confluency conditions may lead to a slight overestimate of the number of AuNPs, and hence the temperature increase, in those dishes which had high local confluency. Since those are the experiments yielding the highest temperatures in Figure 5, where all cells are found to be killed upon irradiation, this does not affect the main conclusion.

sumed to have a heat transfer coefficient of 5 W K^{-1} m^{-2}, although this had no significant effect and essentially the same results were obtained with no heat transfer to air; the dish base and wall were assumed to be thermally insulating. Time steps and spatial simulation cell sizes were chosen to be small enough so that further reduction did not affect the results.

Figure S15 shows a typical example for the increase of temperature upon starting the irradiation, in this case at the maximum intensity available to us, for a cell culture which absorbs 0.3% of the incident light. This absorbance is typical for a HeLa cell culture grown to ~50-80% confluency and incubated with citrate stabilized AuNPs for 3 hours, in agreement with the UV-vis absorbance spectra of cell cultures shown in section S2.3. Since irradiation is carried out at room temperature, this shows that for these incubation conditions it is in fact impossible to reach temperatures above 37°C, which is the normal incubation temperature for HeLa cells. However, much higher temperatures can indeed be achieved by longer incubation with citrate stabilized AuNPs or by incubation with CALNN-AuNPs (see Figure 5 in the main text), which result in the uptake of significantly more AuNPs. Due to the linearity of the Fourier equation, the shape of the spatio-temporal temperature distribution is the same for all experiments, although the amplitude of the temperature change scales with the amount of heat absorbed, i.e. with the laser intensity applied and the density of AuNPs in the irradiated cell culture area.

It is worth noting that for the laser beam diameter used here most of the temperature increase in the center of the laser beam is achieved in the initial 30 seconds, followed by a slower phase which is characterized by the heat spreading into the buffer volume, and thus also increasing the area of the cell culture experiencing a significant temperature increase, without a large temperature increase in the center. This biphasic temperature increase is similar to experimentally observed temperature rises within tumors subjected to AuNP-induced PTT.[33-36]

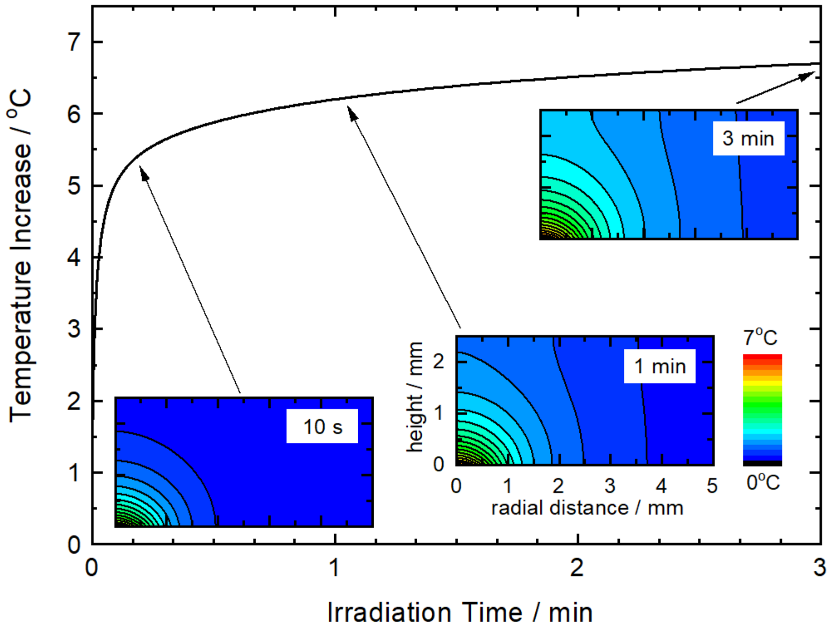

Figure S15. Overall heating of sample. (Main) Time-dependent temperature increase of the HeLa cell monolayer at the center of a laser beam with 210 W/cm^2 intensity and 1.85 mm diameter (1/e^2), for a situation where 0.3% of the incident light is absorbed. (Insets) Contour plots of the spatial temperature distribution in the buffer layer above the HeLa cells after different times of irradiation; horizontal axis: radial distance from the center of the laser beam; vertical axis: height above the cell layer; for clarity, the horizontal and vertical scales as well as the color (temperature) scale are shown only for the distribution after 1 min irradiation, the same scales were used for all plots.

S7. Time Course of HeLa Cell Death after Irradiation in Presence of AuNPs

The standard protocol for determining cell viability was to apply trypan blue 24 hours after irradiation, as described above. In a few experiments, trypan blue was applied at earlier times, washed away and the cells imaged in the microscope, using the same protocol as described above. The cells were then placed under CCM and returned to the incubator to investigate the time course of cell death.

S7.1. PTT Conditions

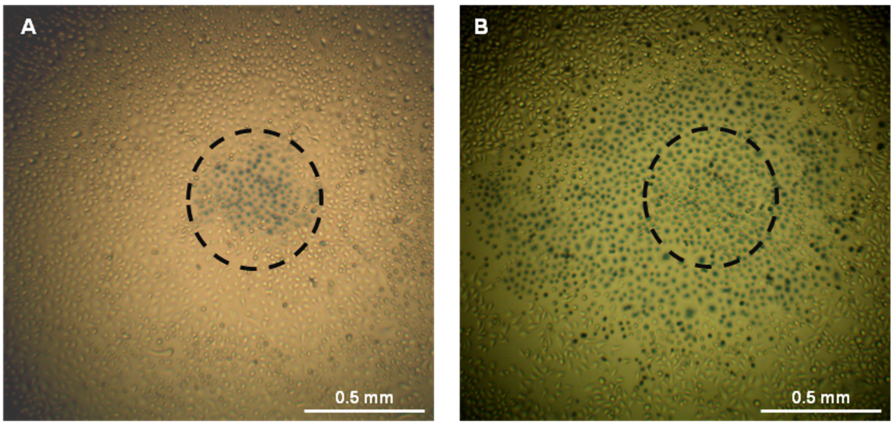

Figure S16. Cell viability determined using the trypan blue assay immediately (A) and 24 hours (B) after irradiation of HeLa cells incubated with 14 nm citrate-stabilized AuNPs (2 nM, 24 hours) at 210 W/cm^2 for 3 minutes. The black dashed circle highlights the cells that died during or immediately after irradiation.

S7.2. PDT Conditions

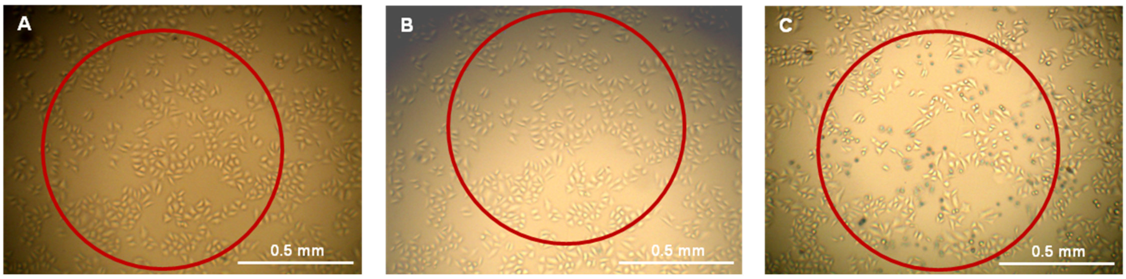

Figure S17. Cell viability determined using the trypan blue assay 10 minutes (A), 30 minutes (B) and 24 hours (C) after irradiation of HeLa cells incubated with 13 nm citrate-stabilized AuNPs (2 nM, 3 hours) at 105 W/cm^2 for 5 minutes. The red circle shows the position of the laser beam.

S8. Investigation of HeLa Cell Killing upon Irradiation in Presence of AuNPs Using the MTT Assay

S8.1. Experimental Details

MTT (3-(4,5-Dimethylthiazol-2-yl)-2,5-Diphenyltetrazolium Bromide), dimethyl sulfoxide (DMSO) and Dulbecco's phosphate buffered saline (Mg2+, Ca2+, glucose, pyruvate) (DPBS) (pH 7.4) were purchased from ThermoFisher. Corning cell culture plates were purchased from Fisher Scientific.

HeLa cells were grown under the conditions described in section S1.4. above in four wells of a 96-well plate (well diameter 6 mm) after seeding with 4000 or 8000 HeLa cells (for 4 or 3 day experiments, respectively). 4 nM citrate-stabilized AuNP solution was mixed with DMEM (1:1, DMEM:AuNP solution) and left standing for 24 hours. Then the DMEM in the wells was replaced in all wells with the AuNP/DMEM mixture or (for control experiments) diluted DMEM (1:1 DMEM:milli-Q H_2O), which was left for either 3 or 24 hours. Following the incubation, cells were washed three times with 100 µL DPBS and then the wells were filled with 300 µL DPBS. One of the four wells was irradiated from below with a cw 532 nm laser beam of 3 W power with a $1/e^2$ diameter of 6 mm, which passed through a 6 mm aperture directly below the well. In order to prevent back-scattered light affecting the control wells, they were covered during irradiation. Following irradiation, the DPBS was replaced with 100 µL DMEM and the well plate was placed back in the incubator for 20 hours.

For the MTT assay, the DMEM was replaced by 100 µL MTT solution (10% MTT solution of 5 mg/mL, 90% DMEM). After 4 hours the MTT solution was removed and 100 µL DMSO were added. This was left for 20 minutes in the incubator before the absorbance at 595 nm and 620 nm was measured for each well using a plate reader (Molecular Devices F3 Filtermax). Following the standard protocol for the MTT assay, the viability was calculated by dividing the absorbance at 595 nm of the irradiated wells, corrected for the background absorbance (measured at 620 nm), by the average absorbance of the control wells. Thus, the viability after irradiation is referenced to the viability of cells containing the same amount of AuNPs, analogous to the analysis performed for the experiments described in the main text using the trypan blue assay. All errors reported are the standard deviation of a minimum of three repeat experiments.

S8.2. Control Experiments

The viability of HeLa cells after incubation with 2 nM of citrate stabilized AuNPs for 3 hours or 24 hours, leading to the uptake of 45,000 and 352,000 AuNPs/cell, respectively (Table S1 above), was investigated in the absence of irradiation. For this purpose, the MTT assay absorbance for cells incubated with AuNP was compared to cells that had been incubated in diluted DMEM for the same time, as described above. The reduction in viability 20 hours after incubation with AuNPs for 3 hours was found to be 0.66 ± 0.10, which is similar to the reduction in cell growth rate observed using the trypan blue assay (see main text). After AuNP incubation for 24 hours the viability was reduced to 0.57 ± 0.08.

The effect of irradiation on HeLa cell viability in the absence of AuNPs was investigated by comparing the MTT assay absorbance for cells that had been irradiated to cells that had not been irradiated, as described above. Irradiation for 3 minutes, 6 minutes or 12 minutes resulted in viabilities of 1.06 ± 0.03, 0.97 ± 0.16 and 0.58 ± 0.08, respectively. For irradiation times under 6 minutes no significant reduction in viability was observed, whereas after irradiation for 12 minutes a significant reduction of was found.

Optical microscopy was used to confirm those results and investigate the effect of washing. Cell cultures that had not been exposed to any washing or irradiation steps but had undergone the same waiting time after seeding as the control samples described here did not show any deformed cells. In contrast, cell cultures that had undergone the washing steps described above,

but had not been exposed to AuNPs or light irradiation, always showed a few deformed/damaged cells, which suggests that the mechanical (shear) forces which the cells experience during washing have some effect on their viability. Therefore, control cells were always subjected to the same washing procedures as the cells undergoing AuNP exposure and/or irradiation. Irradiation for 6 minutes resulted in a few deformed cells, but the extent of this is compatible with the damage observed for cell cultures that had not been exposed to irradiation. In contrast, cell cultures that had been irradiated for 12 minutes showed a significant amount of deformed cells throughout the well, indicating increased cell death due to irradiation.

Simulations analogous to those described above (section S6) confirmed that irradiation under the experimental conditions used here did not result in a temperature rise above 45°C, ruling out any PTT effects.

S8.3. Results

20 hours after incubation of HeLa cells with 2 nM of citrate stabilized AuNPs for 3 hours (resulting in an uptake of 45,000 NPs/cell) and subsequent irradiation at the maximum intensity available (21 W/cm^2 in the center of the laser beam/well) for 6 minutes, the MTT assay showed a viability of these cells of 1.06 ± 0.06 compared to cells that had been incubated with AuNPs but had not been irradiated. In contrast, after incubation for 24 hours (352,000 NPs/cell), irradiation at this intensity and duration resulted in a reduced viability of 0.75 ± 0.07. Given that irradiation of HeLa cells at this intensity for 12 minutes resulted in a measurable reduction of viability already in the absence of AuNPs, we did not attempt to use any longer irradiation times than 6 minutes here.

For the interpretation of these results, one has to take into account that the MTT assay considers the whole cell population in the well under investigation. Therefore, the laser beam had been expanded from a 1/e^2 diameter of 1.9 mm to 6 mm in order to expose as much of the irradiated well to the light as possible and yet achieve significant intensity. However, this still results in a reduction of light intensity at the edge of a 6 mm well by almost 90% compared to the maximum intensity at the well center. This inhomogeneity of the light has a significant effect on the outcome of the experiment and the raw viability data therefore are not directly comparable to the viability data reported for the dish experiments in the main text, where only cells that receive more than 86% of the maximum intensity are taken into consideration.

The cumulative light dose received by the cells in the center of the laser beam under the conditions described above corresponds to the light dose received by the cells in the dish experiments described in the main text using an intensity of 42 W/cm^2 for 3 minutes, although a large fraction receive an even lower dose. Fig. 7 in the main text shows that even a higher dose of 70 W/cm^2 for 3 minutes had no significant effect on cells after incubation with 2 nM AuNPs for 3 hours, compared to the effect of irradiation in the absence of NPs (Table S2). Thus, the result that there is no viability reduction when using the MTT assay on such cells is in agreement with the results obtained on dishes using the trypan blue assay.

More importantly, it is possible to estimate the minimum cumulative light intensity required for killing HeLa cells after incubation with 2 nM AuNPs for 24 hours from the reduced viability of 0.75 ± 0.07 observed after 6 minutes irradiation with a maximum intensity, I_o, of 21 W/cm^2. This estimate takes into account the Gaussian beam profile of the laser intensity $I(r)$,

$$I(r) = I_o e^{-\frac{2r^2}{(d/2)^2}} \qquad \text{(S2)},$$

where r is the distance from the center of the beam/well, I_o is the maximum laser intensity, d is the 1/e^2 diameter of the laser beam (here 6 mm). If the threshold for killing cells is given by a laser intensity T, it is straightforward to show that the fraction f of the total well area (diameter w, here 6 mm) that is exposed to an intensity higher than T is given by

$$f = \frac{d^2}{2w^2} \ln\left(\frac{I_o}{T}\right) \quad \text{(S3).}$$

Assuming that the cells are evenly distributed over the well area, this fraction of the well area which is exposed to a light intensity higher than *T* is complementary to the observed viability, i.e. the viability is given by 1-*f*. Based on Eq. (S3), the observed viability of 0.75 results in a threshold intensity for killing, *T*, of 13 W/cm^2, which corresponds to a cumulative energy incident on each cell during 6 minutes of irradiation of 0.046 J, assuming a typical cell area of 1000 μm^2. These experiments had been conducted after incubation of the HeLa cells with 2 nM AuNPs for 24 hours, resulting in an uptake of 352,000 AuNPs/cell. Since the amount of $^1O_2$ generated upon irradiation of AuNPs is proportional to the number of AuNPs present, this suggests a threshold for killing of 0.36 J incident on each cell for experiments after 3 hours incubation, when only 45,000 AuNPs are present per cell. This is in very good agreement with the threshold of 0.4 J found from dish experiments using the trypan blue assay, see the main text and Figure 7.

It should be noted that the MTT assay approach here is limited. Whereas experiments using the trypan blue assay on cell dishes could easily reach higher cumulative irradiation doses, this is not possible with the MTT assay due to the need of irradiating the whole well. The control experiments also show that it is not possible to extend the duration of the light exposure without affecting cells even in the absence of AuNPs. Shear forces created during washing seem to also affect cell viability, unlike washing in larger dishes where the removal/addition of liquid can be performed far from the cells under investigation. Simulations of the temperature effects analogous to those described in Section S6 above furthermore suggest the potential for more heating in these experiments, since the well geometry prevents the efficient removal of heat energy unlike the situation in the much larger dishes, although we want to highlight that the temperature did not exceed 45°C in our experiments, which is the lower limit for PTT effects, see Figure 5 in the main text.

References

(1) Frens, G. Controlled Nucleation for the Regulation of the Particle Size in Monodisperse Gold Suspension. *Nature Phys. Sci.* **1973**, *241*, 20-22. DOI: 10.1038/physci241020a0.

(2) Levy, R.; Thanh, N. T. K.; Doty, R. C.; Hussain, I.; Nichols, R. J.; Schiffrin, D. J.; Brust, M.; Fernig, D. G. Rational and Combinatorial Design of Peptide Capping Ligands for Gold Nanoparticles. *J. Am. Chem. Soc.* **2004**, *126*, 10076-10084. DOI: 10.1021/ja0487269.

(3) Krpetic, Z.; Davidson, A. M.; Volk, M.; Levy, R.; Brust, M.; Cooper, D. L. High-Resolution Sizing of Monolayer-Protected Gold Clusters by Differential Centrifugal Sedimentation. *ACS Nano* **2013**, *7* (10), 8881-8890. DOI: 10.1021/nn403350v.

(4) Davidson, A. M.; Brust, M.; Cooper, D. L.; Volk, M. Sensitive analysis of protein adsorption to colloidal gold by differential centrifugal sedimentation. *Anal. Chem.* **2017**, *89*, 6807-6814. DOI: 10.1021/acs.analchem.7b01229.

(5) Chithrani, B. D.; Ghazani, A. A.; Chan, W. C. W. Determining the size and shape dependence of gold nanoparticle uptake into mammalian cells. *Nano Lett.* **2006**, *6* (4), 662-668. DOI: 10.1021\nl052396o.

(6) Casals, E.; Pfaller, T.; Duschl, A.; Oostingh, G. J.; Puntes, V. Time Evolution of the Nanoparticle Protein Corona. *ACS Nano* **2010**, *4* (7), 3623-3632. DOI: 10.1021/nn901372t.

(7) Maiorano, G.; Sabella, S.; Sorce, B.; Brunetti, V.; Malvindi, M. A.; Cingolani, R.; Pompa, P. P. Effects of cell culture media on the dynamic formation of protein-nanoparticle complexes and influence on the cellular response. *ACS Nano* **2010**, *4* (12), 7481-7491. DOI: 10.1021\nn101557e.

(8) Piella, J.; Bastus, N. G.; Puntes, V. Size-dependent protein-nanoparticle interactions in citrate-stabilized gold nanoparticles: the emergence of the protein corona. *Bioconjugate Chem.* **2017**, *28*, 88-97. DOI: 10.1021/acs.bioconjchem.6b00575.

(9) Lee, C.-W.; Lin, E.-H.; Cheng, J.-Y.; Wei, P.-K. Study of gold nanoparticles and live cells interactions by using planar evanescent wave excitation. *J. Biomed. Opt.* **2009**, *14* (2), 021005. DOI: 10.1117/1.3116710.

(10) Walkey, C. D.; Olsen, J. B.; Guo, H.; Emili, A.; Chan, W. C. W. Nanoparticle size and surface chemistry determine serum protein adsorption and macrophage uptake. *J. Am. Chem. Soc.* **2012**, *134* (4), 2139-2147. DOI: 10.1021/ja2084338.

(11) Nativo, P.; Prior, I. A.; Brust, M. Uptake and intracellular fate of surface-modified gold nanoparticles. *ACS Nano* **2008**, *2* (8), 1639-1644. DOI: 10.1021/nn800330a.

(12) Fleischer, C. C.; Kumar, U.; Payne, C. K. Cellular binding of anionic nanoparticles is inhibited by serum proteins independent of nanoparticle composition. *Biomater. Sci.* **2013**, *1* (9), 975-982. DOI: 10.1039/c3bm60121h.
(13) Hao, X.; Wu, J.; Shan, Y.; Cai, M.; Shang, X.; Jiang, J.; Wang, H. Caveolae-mediated endocytosis of biocompatible gold nanoparticles in living Hela cells. *J. Phys.: Condens.Matter* **2012**, *24*, 164207. DOI: 10.1088/0953-8984/24/16/164207.
(14) Chithrani, B. D.; Chan, W. C. W. Elucidating the mechanism of cellular uptake and removal of protein-coated gold nanoparticles of different sizes and shapes. *Nano Lett.* **2007**, *7* (6), 1542-1550. DOI: 10.1021/nl070363y.
(15) Krpetic, Z.; Nativo, P.; See, V.; Prior, I. A.; Brust, M.; Volk, M. Inflicting controlled nonthermal damage to subcellular structures by laser-activated gold nanoparticles. *Nano Lett.* **2010**, *10*, 4549-4554. DOI: 10.1021/nl103142t.
(16) Francia, V.; Yang, K.; Deville, S.; Reker-Smit, C.; Nelissen, I.; Salvati, A. Corona composition can affect the mechanisms cells use to internalize nanoparticles. *ACS Nano* **2019**, *13*, 11107-11121. DOI: 10.1021/acsnano.9b03824.
(17) Schneider, C. A.; Rasband, W. S.; Eliceiri, K. W. NIH Image to ImageJ: 25 years of image analysis. *Nat. Methods* **2012**, *9*, 671-675. DOI: 10.1038/nmeth.2089.
(18) Puliafito, A.; Hufnagel, L.; Neveu, P.; Streichan, S.; Sigal, A.; Fygenson, D. K.; Shraiman, B. I. Collective and single cell behavior in epithelial contact inhibition. *Proc. Natl. Acad. Sci. U. S. A.* **2012**, *109* (3), 739-744. DOI: 10.1073/pnas.1007809109.
(19) Wang, X.; Hu, X.; Li, J.; Russe, A. C. M.; Kawazoe, N.; Yang, Y.; Chen, G. Influence of cell size on cellular uptake of gold nanoparticles. *Biomater. Sci.* **2016**, *4*, 970-978. DOI: 10.1039/c6bm00171h.
(20) Khetan, J.; Shahinuzzaman, M.; Barua, S.; Barua, D. Quantitative analysis of the correlation between cell size and cellular uptake of particles. *Biophys. J.* **2019**, *116* (347-359). DOI: 10.1016/j.bpj.2018.11.3134.
(21) Shahinuzzaman, M.; Barua, D. Dissecting particle uptake heterogeneity in a cell population using Bayesian Analysis. *Biophys. J.* **2020**, *118*, 1526-1536. DOI: 10.1016/j.bpj.2020.01.043.
(22) Jeynes, J. C. G.; Jeynes, C.; Merchant, M. J.; Kirkby, K. J. Measuring and modelling cell-to-cell variation in uptake of gold nanoparticles. *Analyst* **2013**, *138*, 7070-7074. DOI: 10.1039/c3an01406a.
(23) Khan, J. A.; Pillai, B.; Das, T. K.; Singh, Y.; Maiti, S. Molecular effects of uptake of gold nanoparticles in HeLa cells. *ChemBioChem* **2007**, *8*, 1237-1240. DOI: 10.1002/cbic.200700165.
(24) Mironava, T.; Hadjiargyrou, M.; Simon, M.; Jurukovski, V.; Rafailovich, M. H. Gold nanoparticles cellular toxicity and recovery: effect of size, concentration and exposure time. *Nanotoxicology* **2010**, *4* (1), 120-137. DOI: 10.3109/17435390903471463.
(25) Keblinski, P.; Cahill, D. G.; Bodapati, A.; Sullivan, C. R.; Taton, T. A. Limits of localized heating by electromagnetically excited nanoparticles. *J. Appl. Phys.* **2006**, *100*, 054305. DOI: 10.1063/1.2335783.
(26) Elliott, A. M.; Stafford, R. J.; Schwartz, J.; Wang, J.; Shetty, A. M.; Bourgoyne, C.; O'Neal, P.; Hazle, J. D. Laser-induced thermal response and characterization of nanoparticles for cancer treatment using magnetic resonance thermal imaging. *Med. Phys.* **2007**, *34* (7), 3102-3108. DOI: 10.1118/1.2733801.
(27) Cheong, S.-K.; Krishnan, S.; Cho, S. H. Modeling of plasmonic heating from individual gold nanoshells for near-infrared laser-induced thermal therapy. *Med. Phys.* **2009**, *36* (10), 4664-4671. DOI: 10.1118/1.3215536.
(28) Haiss, W.; Thanh, N. T. K.; Aveyard, J.; Fernig, D. G. Determination of size and concentration of gold nanoparticles from UV-vis spectra. *Anal. Chem.* **2007**, *79*, 4215-4221. DOI: 10.1021/ac0702084.
(29) Jain, P. K.; Lee, K. S.; El-Sayed, I. H.; El-Sayed, M. A. Calculated Absorption and Scattering Properties of Gold Nanoparticles of Different Size,Shape, and Composition: Applications in Biological Imaging and Biomedicine. *J. Phys. Chem. B* **2006**, *110*, 7238-7248. DOI: 10.1021/jp057170o.
(30) Hu, M.; Hartland, G. V. Heat dissipation for Au particles in aqueous solution: Relaxation time versus size. *J. Phys. Chem. B* **2002**, *106*, 7029-7033. DOI: 10.1021/jp020581+.
(31) Koschmieder, E. L. Benard cells and Taylor vortices; Cambridge University Press: New York, 1993.
(32) Heitz, W. L.; Westwater, J. W. Critical Rayleigh numbers for natural convection of water confined in square cells with L/D from 0.5 to 8. *J. Heat Transfer* **1971**, *93*, 188-195. DOI: 10.1115/1.3449783.
(33) Hirsch, L. R.; Stafford, R. J.; Bankson, J. A.; Sershen, S. R.; Rivera, B.; Price, R. E.; Hazle, J. D.; Halas, N. J.; West, J. L. Nanoshell-mediated near-infrared thermal therapy of tumors under magnetic resonance guidance. *Proc. Natl. Acad. Sci. U. S. A.* **2003**, *100* (23), 13549-13554. DOI: 10.1073/pnas.2232479100.
(34) Dickerson, E. B.; Dreaden, E. C.; Huang, X.; El-Sayed, I. H.; Chu, H.; Pushpanketh, S.; McDonald, J. F.; El-Sayed, M. A. Gold nanorod assisted near-infrared plasmonic photothermal therapy (PPTT) of squamous cell carcinoma in mice. *Cancer Lett.* **2008**, *269*, 57-66. DOI: 10.1016/j.canlet.2008.04.026.
(35) Song, J.; Yang, X.; Jacobson, O.; Huang, P.; Sun, X.; Lin, L.; Yan, X.; Niu, G.; Ma, Q.; Chen, X. Ultrasmall gold nanorod vesicles with enhanced tumor accumulation and fast excretion from the body for cancer therapy. *Adv. Mater.* **2015**, *27* (4910-4917). DOI: 10.1002/adma.201502486.

(36) Li, Z.; Huang, H.; Tang, S.; Li, Y.; Yu, X.-F.; Wang, H.; Li, P.; Sun, Z.; Zhang, H.; Liu, C.; Chu, P. K. Small gold nanorods laden macrophages for enhanced tumor coverage in photothermal therapy. *Biomaterials* **2016**, *74*, 144-154. DOI: 10.1016/j.biomaterials.2015.09.038.
